# Genome-wide association study of seed protein, oil and amino acid contents in soybean from maturity groups I to IV

**DOI:** 10.1007/s00122-019-03304-5

**Published:** 2019-02-26

**Authors:** Sungwoo Lee, Kyujung Van, Mikyung Sung, Randall Nelson, Jonathan LaMantia, Leah K. McHale, M. A. Rouf Mian

**Affiliations:** 1 Department of Crop and Soil Sciences, North Carolina State University, Raleigh, NC 27695 USA; 2 Department of Crop Science, Chungnam National University, Daejeon, 34134 South Korea; 3 Department of Horticulture and Crop Science, The Ohio State University, Columbus, OH 43210 USA; 4 Department of Crop Sciences, University of Illinois and USDA-ARS, Urbana, IL 61801 USA; 5 Corn, Soybean Wheat Quality Research Unit, USDA-ARS, Wooster, OH 44691 USA; 6 Center for Soybean Research and Center of Applied Plant Sciences, The Ohio State University, Columbus, OH 43210 USA; 7 Soybean and Nitrogen Fixation Unit, USDA-ARS, Raleigh, NC 27607 USA

## Abstract

**Key message:**

Genomic regions associated with seed protein, oil and amino acid contents were identified by genome-wide association analyses. Geographic distributions of haplotypes indicate scope of improvement of these traits.

**Abstract:**

Soybean [*Glycine max* (L.) Merr.] protein and oil are used worldwide in feed, food and industrial materials. Increasing seed protein and oil contents is important; however, protein content is generally negatively correlated with oil content. We conducted a genome-wide association study using phenotypic data collected from five environments for 621 accessions in maturity groups I–IV and 34,014 markers to identify quantitative trait loci (QTL) for seed content of protein, oil and several essential amino acids. Three and five genomic regions were associated with seed protein and oil contents, respectively. One, three, one and four genomic regions were associated with cysteine, methionine, lysine and threonine content (g kg^−1^ crude protein), respectively. As previously shown, QTL on chromosomes 15 and 20 were associated with seed protein and oil contents, with both exhibiting opposite effects on the two traits, and the chromosome 20 QTL having the most significant effect. A multi-trait mixed model identified trait-specific QTL. A QTL on chromosome 5 increased oil with no effect on protein content, and a QTL on chromosome 10 increased protein content with little effect on oil content. The chromosome 10 QTL co-localized with maturity gene *E2*/*GmGIa*. Identification of trait-specific QTL indicates feasibility to reduce the negative correlation between protein and oil contents. Haplotype blocks were defined at the QTL identified on chromosomes 5, 10, 15 and 20. Frequencies of positive effect haplotypes varied across maturity groups and geographic regions, providing guidance on which alleles have potential to contribute to soybean improvement for specific regions.

**Electronic supplementary material:**

The online version of this article (10.1007/s00122-019-03304-5) contains supplementary material, which is available to authorized users.

## Introduction

Soybean [*Glycine max* (L.) Merr.] is a highly valued source of protein and oil for feed, food and industrial uses across the world. Soybean seed is composed of 35% protein and 18% oil on a 13% moisture basis (Wilson 2004). In 2016, soybean represented 71% of protein meal used worldwide (SoyStat 2017). With defatted soybean meal serving as the main source of protein in the feed mixtures of commercial poultry, livestock and fish farms worldwide, there is a growing interest in elevating the protein content of soybean seed and, in turn, elevating the protein content of the meal obtained from seed (Gatrell et al. 2014). While increasing seed protein content of soybean cultivars has been a major objective of many soybean breeding programs for decades, the negative correlations of seed protein content with seed oil content and seed yield have hampered progress (Burton 1987; Rinker et al. 2014; Patil et al. 2017). Oil also represents a valuable fraction of the soybean seed, with approximately 29% of worldwide production of oil and fat dependent on soybean (SoyStat 2017). Despite the importance of both oil and protein contents, yield remains the ultimate driver of soybean cultivar selection to soybean breeders (Sebastian et al. 2010; Fox et al. 2015; Patil et al. 2017). Nearly 95% of the soybean grown in the USA is commodity soybean for which farmers are paid by weight and not composition. Consequently, in cultivar development, breeders generally select for the best seed yield potential with little attention to seed protein or oil content. Since seed protein contents of US soybean cultivars have been declining slowly over many decades, while the seed yields have been increasing (Rinker et al. 2014), developing high protein and high yield soybean cultivars would benefit farmers (Chung et al. 2003; Bandillo et al. 2015; Brzostowski and Diers 2017).

Currently, over 300 quantitative trait loci (QTL) associated with protein content in soybean from many studies have been reported (http://www.soybase.org; Van and McHale 2017). Diers et al. (1992) identified a major QTL for protein on chromosome (Chr) 20. This QTL region has been repeatedly identified in a number of studies and a more precise genomic location has been determined by sequence-based molecular mapping studies (Sebolt et al. 2000; Specht et al. 2001; Chung et al. 2003; Nichols et al. 2006; Jun et al. 2008; Rodrigues et al. 2010; Yan et al. 2014; Warrington et al. 2015). The genomic location of this QTL was narrowed to *a* < 1 Mb window based on genome-wide association analysis with large-scale genotypic and phenotypic data (Vaughn et al. 2014). Bandillo et al. (2015) further narrowed the Chr 20 region to encompass only three possible candidate genes in *G. max* reference genome (Glyma.Wm82.a1.v1.1, 1.1 gigabase, http://www.soybase.org). A Korean soybean cultivar ‘Danbaekkong’ (PI 619083) (Kim et al. 1996) is a well-studied source of a high protein allele at the Chr 20 QTL. The Danbaekkong allele accounted for 55% of phenotypic variation in protein content in a Benning × Danbaekkong population, and this high protein allele of Danbaekkong has been used as a source to increase crude protein content in US soybean cultivar development (Warrington et al. 2015; Brzostowski et al. 2017; Mian et al. 2017). This high protein Danbaekkong allele has a strong negative effect on seed oil content, resulting in a 1% reduction of oil content for every 2% increase in protein content (Bandillo et al. 2015; Warrington et al. 2015; Patil et al. 2017).

The nutritional value of soybean protein is also important in the production of poultry and livestock, but can be limited by key essential amino acids. Methionine (Met) and cysteine (Cys) are the two sulfur-containing amino acids that are important for poultry production. Met is the initiating amino acid in eukaryotic protein synthesis, and Cys is critical in the formation of disulfide bonds (Brosnan and Brosnan 2006). Threonine (Thr) and lysine (Lys) are also generally deficient in soybean meal (Warrington et al. 2015; Patil et al. 2017). Unlike protein, only a few studies investigating the genetic control of variation in the amino acid content of soybean have been published (Panthee et al. 2006; Vaughn et al. 2014; Warrington et al. 2015). However, many QTL related to the 7S (β-conglycinin) and 11S (glycinin) fractions of soybean storage proteins have been identified on Chrs 1, 3, 4, 6, 10, 13, 16, 17, 19 and 20 (Panthee et al. 2004; Ma et al. 2016; Boehm Jr. et al. 2018). The glycinin fraction contains higher levels of S-containing amino acids than the β-conglycinin fraction (Warrington et al. 2015).

Modern plant breeding has resulted in genetic erosion in many cultivated crop species (e.g., Gizlice et al. 1994; Hufford et al. 2012; Lam et al. 2010). Identification of useful genetic diversity is expected to provide great opportunities to improve traits of interest in the future. At present, nearly 20,000 introduced accessions of the genus *Glycine* are available from USDA Soybean Germplasm Collection (GRIN, http://www.ars-grin.gov/cgi-bin/npgs/html/crop.pl?51), but only a limited number of accessions have been extensively evaluated for seed composition traits. The SNP data of 19,652 accessions assayed with SoySNP50K iSelect BeadChip (Song et al. 2015) are publicly available and have been used to investigate genomic diversity and population structure of the large collection of germplasm (Jarquin et al. 2016; Valliyodan et al. 2016). This set of SNP data has also been utilized in genome-wide association studies (GWAS) to dissect quantitative traits based on historical recombination and genetic variation within this wide diversity of germplasm (Bandillo et al. 2015; Hwang et al. 2014; Vaughn et al. 2014). Seed protein and oil contents have been among the traits examined using GWAS and the USDA soybean germplasm genotyped with the SoySNP50K iSelect BeadChip. However, these earlier studies have used protein and oil content data from GRIN collected from fields with a single replicate of incomplete sets of lines (Bandillo et al. 2015; Vaughn et al. 2014) or have relied on relatively small populations (Hwang et al. 2014).

In the present study, a total of 621 soybean accessions in maturity groups (MGs) I to IV (Zhang et al. 2007) were used as a GWAS panel. Field trials for seed composition with this panel were conducted in five different environments (a single location in 2014 and four locations and multi-state trials in 2015). The multi-location phenotypic data for seed composition, which included protein, oil and four essential amino acids, were analyzed with the 34,014 SNP markers from the SoySNP50K iSelect BeadChip to dissect the genomic architecture of these traits through GWAS.

## Materials and methods

### Plant materials and seed samples

A collection of 877 accessions of *G. max* (2*n* = 40) in MGs I to IV from the USDA Soybean Germplasm Collection were selected to represent maximum variations in seed protein content and seed oil content according to data extracted from GRIN. In addition, only accessions with yellow seed-coat color and < 4 scores (on a 1 to 5 scale) for lodging, pod shattering and seed mottling (with few exceptions) were considered. In 2014, the 877 accessions were planted in 3.7 m single row plots in a field in Wooster, OH (OHW14) including four blocks of 200 entries and a block of 87 with a set of four checks entered at the beginning of each block. Agronomic data (e.g., days to maturity, lodging and seed shattering) were also collected, and rows were harvested. Based mainly on the agronomic data, 621 of the 877 accessions were advanced for further evaluation. Accessions that matured earlier than September 21 or later than October 25, 2014 or had lodging scores of > 4 on a scale of 1–5 (Fehr and Caviness 1977) were discarded. In addition, lines having fewer than 500 high-quality seed were not included. In 2015, the 621 accessions (Table S1) were planted in three mid-western and one southern locations of the USA [Wooster (OHW15) and Columbus, OH (OHC15), Urbana, IL (IL15) and Plymouth, NC (NC15)] with two replications at each environment in an augmented randomized complete block design (ARCBD). Each replicate within the ARCBD was divided into 4 blocks, which included no more than 196 accessions and four checks: ‘Summit’ (McHale et al. 2013), ‘Wyandot’ (Lee et al. 2017; https://mchalelab.cfaes.ohio-state.edu/sites/mchale/files/imce/Wyandot_release_document.pdf), HR09-397 (a high protein breeding line from USDA-ARS, Wooster, OH) and ‘Prohio’ (Mian et al. 2008).

### Determinations of protein, oil and amino acid contents

All seed samples were cleaned by eliminating molded, mottled, discolored or off-type seeds. The dry-matter-based protein and oil contents in whole seed were measured by a DA 7250 Near Infrared Analyzer spectrometer (NIRS) (Perten Instruments^®^, Hägersten, Sweden), and each value was converted to a 13% moisture basis. For the NIRS calibration, the annually updated manufacturer’s calibration module was used. The selected amino acid contents were also measured simultaneously and reported on a g kg^−1^ crude protein (cp) basis.

Determination of amino acids contents by destructive method was according to Warrington et al. (2015). The 80-g whole seed samples were ground using a Perten Laboratory Mill 3610 grinder (Perten Instruments^®^, Hägersten, Sweden). Ground samples were scanned by NIRS. The overall Pearson’s correlation coefficients (*r*) of amino acid values between whole and ground samples of the four amino acids—Met, Cys, Lys and Thr—were 0.85, 0.73, 0.91 and 0.79, respectively (Table S2). Due to the high correlation values of each amino acid between ground and whole seed samples, we used the amino acid data from the whole seed for all replicates of all environments for downstream analyses.

### Statistical analysis of phenotypic data

Best linear unbiased predictor (BLUP) values were calculated from multi-environment phenotype data using PROC MIXED (SAS Institute 2013). Phenotypic data included protein and oil content on a g kg^−1^ basis and amino acid content on a g kg^−1^ crude protein basis. The estimated BLUP values represent relative genetic values of individual genotypes within the collection of accessions, obtained by partially excluding non-genetic effects on a given trait with the following statistical model:

$$Y_{ijklm} = \mu + E_{i} + R\left( E \right)_{ij} + B\left( {RE} \right)_{ijk} + C_{l} + G\left( C \right)_{lm} + \varepsilon_{ijklm}$$

where *μ* is overall mean, *E*_*i*_ is effect of *i*th environment, *R*(*E*)_*ij*_ is effect of *j*th replication in *i*th environment, *B*(*RE*)_*ijk*_ is effect of *k*th block in *j*th replication in *i*th environment, *C*_*l*_ is effect of *l*th class of entry (*l* = 1, 2, 3, 4 and 5 for four checks, Summit, Wyandot, HR09-397 and Prohio and germplasm accessions, respectively), *G*(*C*)_*lm*_ is effect of *m*th entry within class, and *ε*_*ijklm*_ is experimental error. Class of entry was treated as a fixed effect, and all other terms were treated as random effects. For phenotypic data across all environments (ALL), BLUP values were extracted from the random effect estimates of *G*(*C*)_*lm*_ and scaled by addition of the intercept to each BLUP value. For phenotypic data in individual environments, the same procedures were followed, but *E*_*i*_ was not included in models. Variance components were estimated using PROC VARCOMP (SAS Institute 2013) with the restricted maximum likelihood (REML) method (Patterson and Thompson 1971). With these variance components, the broad-sense heritability (*H*^2^) on an entry-mean basis was calculated for each trait as follows: $\sigma^{2}_{G} / \, \left( {\sigma^{2}_{G} + \sigma^{2}_{GE} /e \, + \sigma^{2} /r} \right)$, where *e* is the number of environment per genotype and *r* is the total number of replication per genotype (i.e., 9).

### Genotypic data of the *G. max* accessions

Publicly available SNP marker data (http://soybase.org/snps/) of the 621 accessions were downloaded from the SoySNP50K SNPs data repository (Song et al. 2013). Initially starting with 42,180 SNPs, 2109 and 5805 SNPs were removed from the dataset due to monomorphism or low minor allele frequency (MAF) (< 0.05), respectively. Seven SNPs with MAF between 0.035 and 0.05 were purposely not excluded because they are located in the previously known QTL region of Chr 20. Seventy and 182 SNPs were additionally eliminated due to higher than 10% of missing genotypes (i.e., undefined genotypes) and undetermined chromosomal position, respectively. After filtering, a total of 34,014 SNPs were used in the GWAS.

### Population structure

The software STRUCTURE version 2.3.1 (Pritchard et al. 2010) and STRAUTO (Chhatre and Emerson 2017) were used to determine population structure among the 621 accessions. The whole set of SNP data was pruned to 3401 SNPs by selecting every tenth from the 34,014 SNPs, which were used to infer ancestry of individuals. Ten independent runs were conducted for each specified *K *= 1 to 9 (number of subpopulation), with 50,000 burn-in period, 1,000,000 Markov chain Monte Carlo (MCMC) iterations. ‘*Delta K*’ method (Evanno et al. 2005) was used to determine the most likely number of clusters via a web-based informatics tool STRUCTURE HARVESTER (Earl and vonHoldt 2012). The method estimates *delta K* based on the rate of change in the log probability between successive *K* values. Replicated runs of STRUCTURE were permutated with the software CLUMPP v1.1 (Jakobsson and Rosenberg 2007) to obtain the *Q* matrix. A bar plot was drawn using the software DISTRUCT (Rosenberg 2004). Principal component analysis was also performed to examine genetic structure and variation in our *G. max* accessions by the origin of country (Table S1).

### Linkage disequilibrium (LD)

Haploview 4.2 (Barrett et al. 2005) was used to calculate correlation coefficient (*r*^2^) of alleles to measure LD with the following criteria: maximum distance as 1000 kb, minimum minor allele frequency as 0.05, and *r*^2^ was calculated pairwise for markers across each chromosome. Genome-wide LD was plotted as physical distance (kb) versus *r*^2^ with smooth curves fitted by LOESS (locally weighted regression) for both euchromatin and heterochromatin. The LD decay to half maximum value and to *r*^2^ = 0.2 were determined based on the LOESS curves. Haplotype blocks were determined using the four-gamete method (Wang et al. 2002) with a Hardy–Weinberg cutoff as *P* value < 0.01. A SNP was not included in the haplotype block if addition of the SNP to the block resulted in a recombinant allele at a frequency exceeding 1%. Adjacent blocks were combined, if these blocks were separated by less than 10 kb (Schneider et al. 2016).

### Genome-wide association analysis

Associations between genotypic and single trait phenotypic data (scaled BLUP values) were investigated with two different models; compressed mixed linear model (CMLM, Zhang et al. 2010) and multi-locus mixed model (MLMM, Segura et al. 2012). For the CMLM analysis, Genome Association Prediction Tool (GAPIT) (Lipka et al. 2012) was used with the optimal number of principal components (PCs) determined by the Bayesian information criterion, a one PC was identified as the best fit for all traits. The CMLM included the predetermined PCs (*P* matrix) and kinship (*K* matrix) calculated by the VanRaden method (VanRaden 2008). A modified version of MLMM, MLMM_cof (https://github.com/Gregor-Mendel-Institute/MultLocMixMod), which allows PCs to be used as covariates, was used as the second approach for evaluating associations. The first three PCs, accounting for a total of 18.3% genotypic variation, were used as covariates. Prior to the MLMM analysis, genotypic data were imputed by TASSEL 5.0 software (Bradbury et al. 2007) with LD *k*-nearest neighbors imputation option (Money et al. 2015).

A multi-trait mixed model (MTMM, Korte et al. 2012) allows improved power in GWAS of correlated traits, such as protein and oil contents in soybean seed. Using the imputed genotype data, several MTMM models were applied to identify genomic regions exhibiting pleiotropy for protein and oil contents, in which they have the expected negative correlation (“opposite effect” alleles), but also to identify loci which effect only one of the two traits (“trait-specific” alleles). Opposite effect loci identified by MTMM were defined as those exhibiting negative pleiotropy, affecting both traits, but in the opposite ways (i.e., an allele with a positive effect on oil content and a negative effect on protein content), whereas trait-specific loci identified by MTMM were defined as those effected one trait, but having little or no effect on the second trait. MTMM scripts are available at https://github.com/Gregor-Mendel-Institute/mtmm. MTMM analysis was performed using a set of R scripts, which are dependent on the software ASReml (Gilmour et al. 2015).

A modified Bonferroni adjustment for multiple testing was applied to calculate the significance threshold for marker-trait associations. The effective number of independent tests (*M*_eff_) calculated by simpleM (Gao et al. 2010, http://simplem.sourceforge.net/) was estimated to be 13,750 from the 34,014 SNPs genotyped over 621 individuals. Thus, the adjusted significance thresholds at α as 5% for genome-wide significance and 10% and 25% for suggestive thresholds were − log_10_ (*P*) > 5.44, − log_10_ (*P*) > 5.14, and − log_10_ (*P*) > 4.74, respectively. Calculation of the correlation matrix and eigenvalue decomposition by simpleM was conducted in *R*. All GWAS analyses were also conducted in an R implementation (http://www.r-project.org). Manhattan and quantile–quantile plots were used to visualize each association (Turner 2014).

## Results

### Phenotypic variations and correlations

**Fig. 1:**
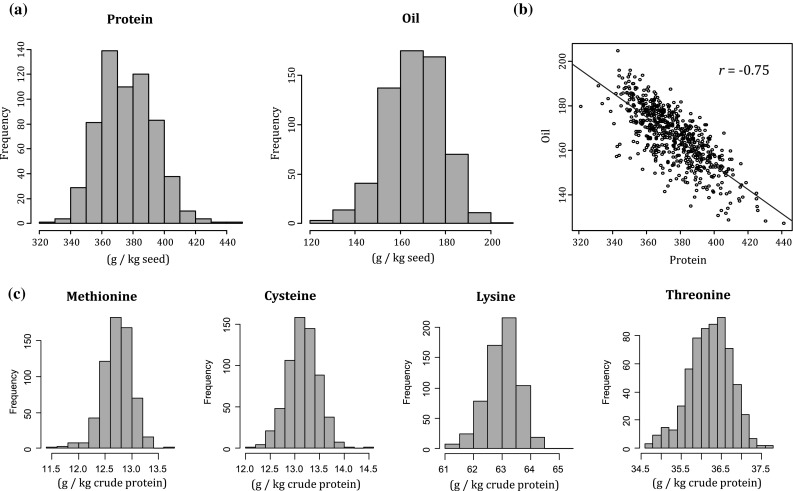
Phenotypic distribution of seed protein and oil contents (**a**) by scaled best linear unbiased predictor (BLUP) values across all environments (ALL) among the 621 plant introductions and their correlation (*P* < 0.0001) (**b**). Phenotypic distribution of amino acids by scaled BLUP values across all environments was also shown (**c**)

Wide and continuous phenotypic distributions were observed for both seed protein and oil contents (mean and scaled BLUP values across all environments) (Fig. 1; Tables S3, S4). Estimates of variance components are given in Table 1. Analysis of variance indicated that protein and oil contents were significantly different among the five environments; OHW14, OHW15, OHC15, IL15 and NC15 (*P* < 0.001; Table S5). Correlation coefficients among individual environments were high, ranging from 0.53 to 0.94 for protein content and from 0.67 to 0.92 for oil content (correlations based on means, Table S6). For ALL environment, scaled BLUP values ranged from 327 to 439 g kg^−1^ for protein content and 129 to 210 g kg^−1^ for oil content (Fig. 1). Average protein content was highest in OHC15 (385 g kg^−1^) and lowest in IL15 (361 g kg^−1^) (Table S4). As reported in numerous previous studies (e.g., Burton 1987; Vaughn et al. 2014; Wilson 2004), a strong negative correlation (*r *= − 0.75; *P* < 0.0001) between seed protein and oil contents was found (Fig. 1b). Broad-sense heritability (*H*^*2*^) was 0.94 and 0.97 for protein and oil contents, respectively (Table 1).

**Table 1:** Variance component estimates and broad-sense heritability of traits assessed in 621 soybean accessions grown at Wooster, OH, in 2014 and 2015 (OHW14, OHW15), Columbus, OH, in 2015 (OHC15), Urbana, IL, in 2015 (IL15) and Plymouth, NC, in 2015 (NC15)

Parameter	Protein (g kg^−1^)	Oil (g kg^−1^)	Methionine (g kg^−1^ cp)	Cysteine (g kg^−1^ cp)	Lysine (g kg^−1^ cp)	Threonine (g kg^−1^ cp)
Environment	0.87	1.05	0.05	0.04	0.12	0.01
Replication (environment)	0.00	0.00	0.02	0.04	0.04	0.02
Block (replication × environment)	0.87	0.24	0.01	0.02	0.05	0.06
Genotype	3.21	1.71	0.08	0.11	0.35	0.28
Genotype × environment	0.56	0.17	0.02	0.05	0.12	0.06
Error	0.97	0.23	0.06	0.16	0.26	0.17
Broad-sense heritability	0.94	0.97	0.88	0.80	0.87	0.90

Each amino acid content on a seed weight basis (means) showed continuous and wide distributions, with overall mean ranging 3.8 to 5.7 g kg^−1^, 3.9 to 6.3 g kg^−1^, 12.9 to 28.4 g kg^−1^ and 11.1 to 16.1 g kg^−1^ for Met, Cys, Lys and Thr, respectively (Table S3). Correlation coefficients of means of each amino acid among individual environments ranged 0.52 to 0.86, 0.53 to 0.81, 0.52 to 0.87 and 0.48 to 0.84 for Met, Cys, Lys and Thr, respectively (Table S6). Amino acid amount on a seed weight basis was strongly correlated with protein levels, with correlation coefficients of 0.89, 0.87, 0.97 and 0.99 for Met, Cys, Thr and Lys, respectively. Because the amount of amino acids on a seed weight basis is highly dependent on the amount of crude protein in the seed, amino acid data were instead presented as the amount (*g*) of a specific amino acid per kg of crude protein (g kg^−1^ cp). Phenotypic distribution of each amino acid (g kg^−1^ cp) based on scaled BLUP values is shown in Fig. 1c. For each of the four amino acids, phenotypes of the 621 entries were normally distributed (Fig. 1c). Sulfur-containing amino acids: Met and Cys contents ranged from 11.5 to 13.7 and 12.1 to 14.5 g kg^−1^ cp, respectively. Compared to Cys and Met, Thr and Lys comprised larger proportion of crude protein, ranging between 34.7 to 37.7 and 61.2 to 65.0 g kg^−1^ cp, respectively (Fig. 1c; Table S7). Met, Thr and Lys on a g kg^−1^ cp basis each had a significant negative correlation to total crude protein (*r* = − 0.58, − 0.82 and − 0.82, respectively; *P* < 0.0001; Fig. S1). Met was moderately correlated with the other three amino acids (*r* = 0.57, 0.64, 0.69 for Cys, Thr and Lys, respectively; *P* < 0.0001) (Table S8). A strong positive correlation (*r* = 0.91; *P* < 0.0001) between Lys and Thr was observed, whereas Cys was only weakly correlated (*r* = 0.12 and 0.14; *P* = 0.01 and 0.001, respectively) with Thr and Lys (Table S8).

### Population structure and linkage disequilibrium

**Fig. 2:**
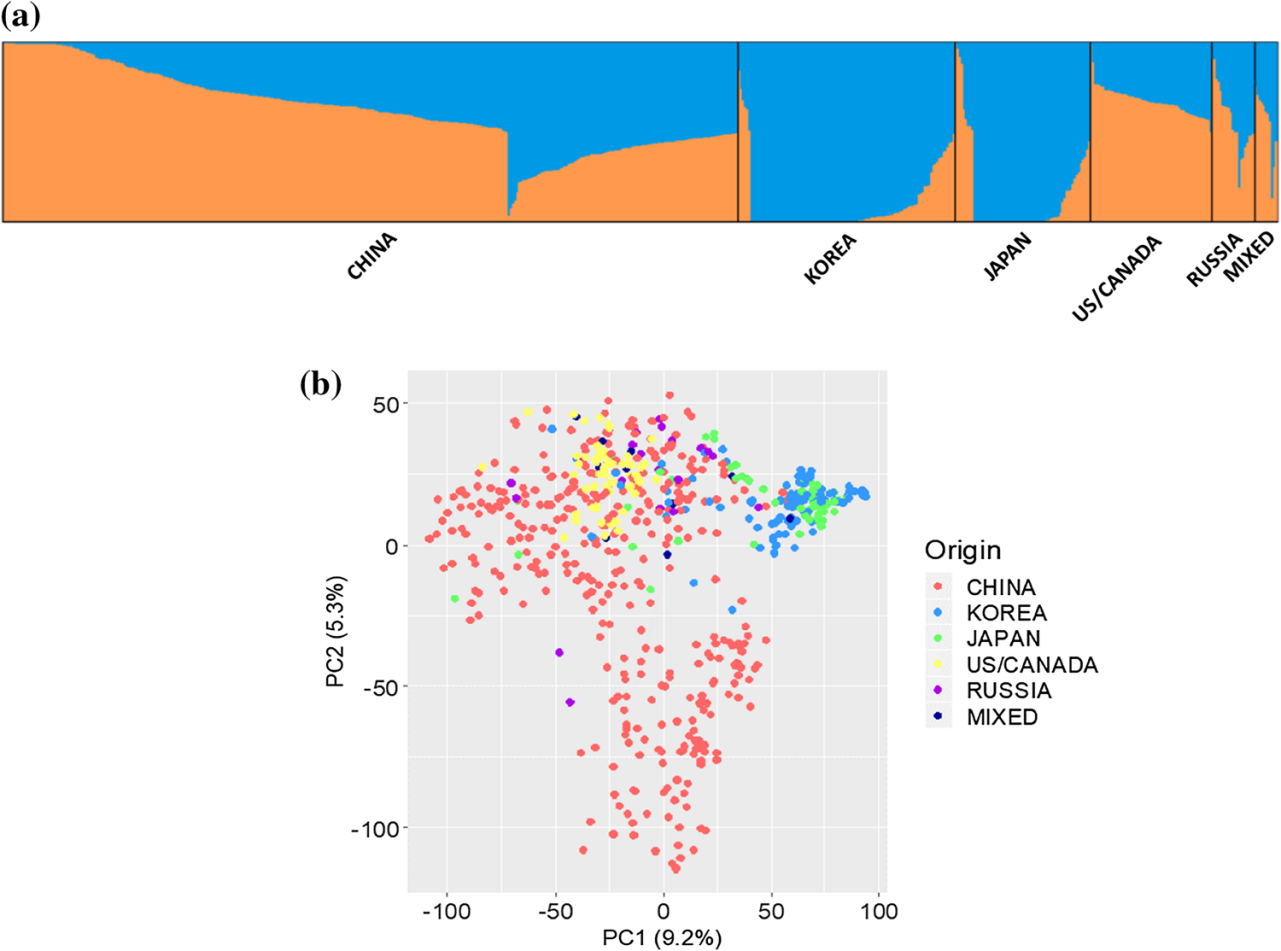
Population structure of the 621 soybean accessions. **a** Plot of STRUCTURE analysis (*K *= 2). Accessions were sorted by geographic location from which each accession was collected, and colored bars correspond to the STRUCTURE assignments (Q1 and Q2) (**b**). Principle component analysis (PCA) of the 621 soybean accessions with the country of origin indicated by color of marker

Population structure was analyzed using the software STRUCTURE 2.4.3. The ‘*Delta K*’ method (Evanno et al. 2005) supported *K *= 2 as the most likely number of subpopulations (Figs. 2a, S2). Principal component analysis was also used to infer population structure (Fig. 2b). The vast majority (~ 95%) of the 621 accessions originated from China, Korea, Japan and USA. Accessions collected from northeastern China (*n* = 246) and US (*n* = 57) were mainly grouped in subpopulation 1. Most Korean (94%) and Japanese (86%) accessions were grouped with one-third of Chinese accession, which predominantly originated in southern China (including Anhui and Shandong provinces).

The squared allele frequency correlation (*r*^2^) was obtained for intra-chromosomal loci pairs (Fig. S3). Across entire chromosomes, the extent of LD for an *r*^2^ of 0.2 varied by chromosome, ranging from 199 to 1015 kb and the extent of LD over the whole genome was approximately 390 kb (Table S9).

### Genome-wide association analyses for protein and oil contents

**Fig. 3:**
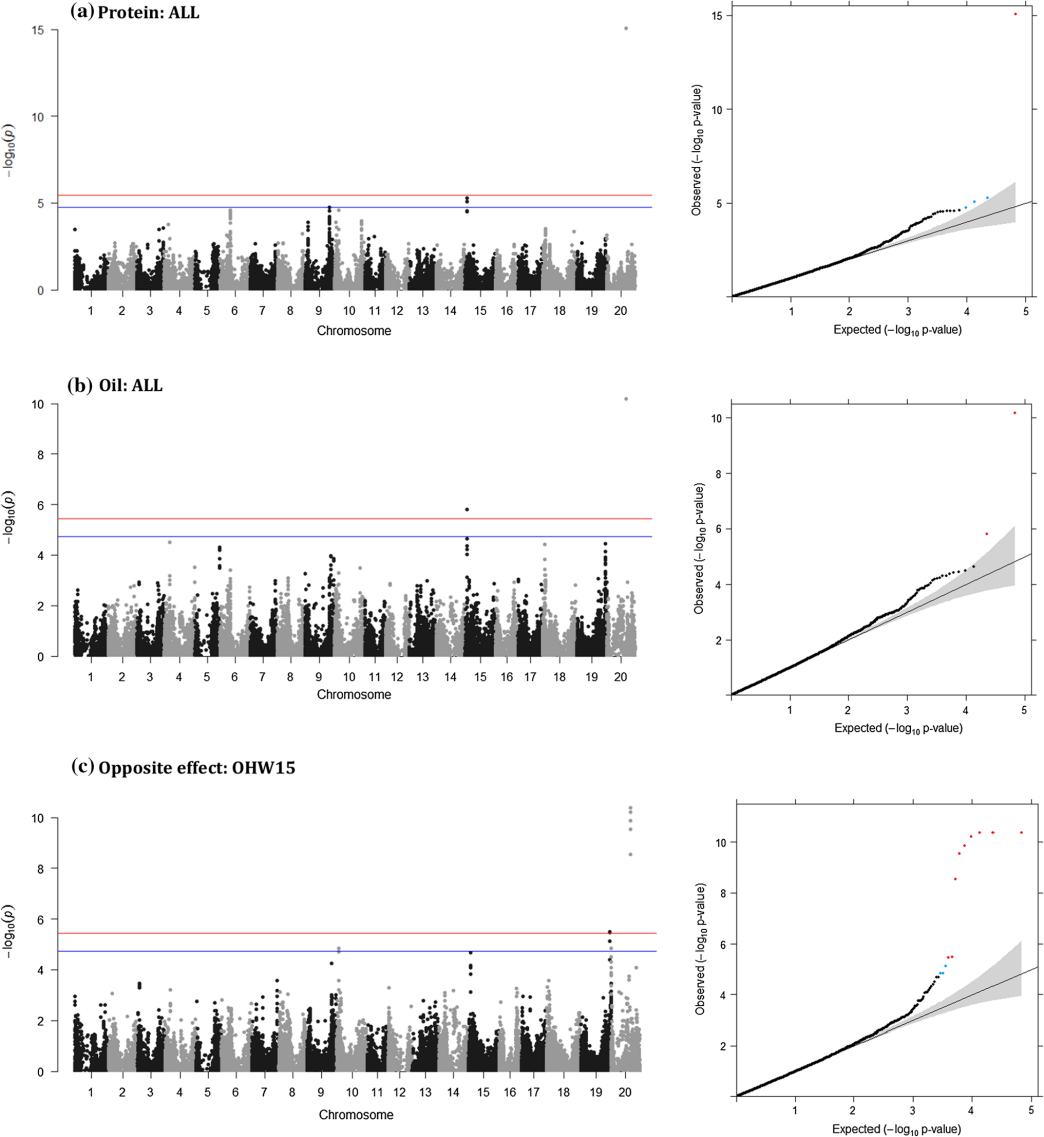
Manhattan plots (left) and QQ-plots (right) for GWAS of the 621 soybean accessions for protein (**a**) and oil (**b**) contents using multi-locus mixed model and opposite effect (**c**) by multi-trait mixed model. The trait associations for 34,014 SNPs were plotted by all environments combined (ALL) (**a** and **b**) or the Wooster, Ohio 2015 environment (OHW15) (**c**). Red and blue horizontal lines in the Manhattan plots and markers in the QQ-plots represent the genome-wide significant threshold (5%) and suggestive significance thresholds (25%), respectively, and the SNPs significantly associated at those levels. Shaded regions of the QQ-plots represent a 95% confidence interval (color figure online)

Association analyses were implemented for protein and oil contents with both CMLM and MLMM models by each environment (OHW14, OHW15, OHC15, IL15 and NC15) and all environments combined (ALL). The qq-plots of both CMLM (Figs. S4, S5), MLMM (Figs. 3a, b, S6, S7) and MTMM (opposite effect) models (Figs. 3c, S8) showed a sharp deviation from the expected *P* value distribution at the tail, indicating that both models effectively controlled for false-positive and false-negative associations.

In total, 16 and 19 SNPs were identified with genome-wide significant or suggestive associations to seed protein and oil contents, respectively, using the CMLM and MLMM models. Twenty-three opposite effect SNPs (loci exhibiting negative pleiotropy for protein and oil, e.g., an allele contributing positively to protein content and negatively to oil content, or vice versa) were identified with the MTMM-opposite effect model. SNPs were assigned significance at adjusted genome-wide significance level of 5% (− log_10_ (*P*) > 5.44), and suggestive thresholds of 10 and 25% (− log_10_ (*P*) > 5.14 and 4.74, respectively). To simplify the presentation of results, only the most significant SNP in each LD block was selected as the representing locus and listed by trait, environment and analytic method (Table S10).

Seventeen LD blocks, represented by the most significant SNPs in each block, were associated with seed protein or oil content at a suggestive threshold (8 SNPs) or genome-wide significance threshold (10 SNPs) identified by either CMLM, MLMM or MTMM-opposite effect and distributed across nine chromosomes (Chrs 4, 5, 8, 9, 10, 13, 15, 19 and 20) (Table S10). Significance of the association was often environmentally dependent (Figs. S9, S10; Table S10). Among the 18 significant SNPs, ten SNPs (ss715589592 on Chr 4, ss715591710 on Chr 5, ss715600889 on Chr 8, ss715606797 on Chr 10, ss715616001 on Chr 13, ss715621799 on Chr 15, ss715635870 on Chr 19 and ss715638960, ss715637217, ss715637225 on Chr 20) displayed significant or suggestive associations with either protein content, oil content, or both (MTMM-opposite effect) in only one of the five environments (excluding ALL). CMLM and MLMM resulted in similar significant associations (Table S10), thus, our reporting of results focuses on MLMM and MTMM-opposite effect associations, which are significant (genome-wide threshold of 5%). Of the 14 LD blocks identified as associated with seed protein or oil content via MLMM or MTMM-opposite effect analyses, seven blocks on three chromosomes (Chrs 15, 19 and 20) possessed significant SNPs at the genome-wide threshold (Table 2).

**Table 2:**
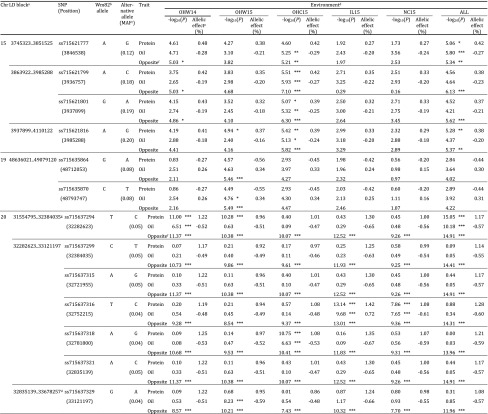
Significant SNPs on chromosomes 15, 19 and 20 associated with protein and oil contents (g kg^−1^ seed) from multi-locus mixed model and multi-trait mixed model-opposite analyses

*, suggestive threshold (25%); **, suggestive threshold (10%); ***, genome-wide significance threshold (5%)

^a^Linkage disequilibrium (LD) blocks were constructed based on four-gamete method. Blocks were merged, if adjacent blocks were separated by < 10 kb

^b^Williams 82

^c^Minor allele frequency

^d^OHW14, Wooster, OH, 2014; OHW15, Wooster, OH, 2015; OHC15, Columbus, OH, 2015; IL15, Urbana, IL, 2015; NC15, Plymouth, NC, 2015; ALL, all environments

^e^Allelic effect of alternative allele relative to Williams 82

^f^Locus associated with an opposite effect for protein and oil identified by multi-trait mixed model

^g^SNP was not in LD with any other SNPS; thus, LD block was defined by positions of adjacent markers

The genomic regions significantly associated with protein and oil contents on Chr 15 grouped into three LD blocks. The most significant SNP representing each block (ss715621777, ss715621799 and ss715621816) was significant only in the OH (OHW14, OHW15 and OHC15) or ALL environments, but not in IL15 or NC15 (Table S10). Four total SNPs within these three Chr 15 LD blocks were identified as significant (*α* = 5%) using at least one model (Table 2). Previous studies have found the effect of Chr 15 loci to be environmentally dependent (Pathan et al. 2013; Tajuddin et al. 2003). In this study, although Chr 15 markers were significant in only Wooster, Ohio environments, there was no significant allele × environment interaction (Table S5). Under ALL environment, the alternative alleles (relative to Williams 82) of the four significant SNPs on average increased protein content by 0.39% and decreased seed oil content by 0.23%, displaying a − 1.7 ratio between protein and oil contents.

The LD block on Chr 19 was identified as significant (*α* = 5%) only in the MTMM-opposite effect analysis, which gains power from the negative correlation between protein and oil contents (Table 2). Both significant SNPs in this LD block, ss715635864 and ss715635870, are only associated at the significant or suggestive level in the MTMM-opposite effect analysis with OHW15 data. The alternative alleles of these two SNPs decreased protein content by 0.55% and increased oil by 0.34%, resulting in a − 1.6 ratio between protein and oil contents.

The seven significantly associated SNPs on Chr 20 fell into three LD blocks (Table 2). In contrast to the Chr 15 and 19 loci, on Chr 20, SNPs associated with these three LD blocks possessed significant associations across a range of environments in the MTMM-opposite effect analysis (Table 2). While each of the four MLMM significant SNPs (ss715637294, ss715637316, ss715637318 and ss715637329) was only significant in one or two environments, most environments were represented by significant SNPs in the MTMM-opposite effect analysis (Table 2). Their alternative allelic effects in protein content (1.18%) and oil content (− 0.58%) were 2.2 and 2.5-fold greater than the effects conferred by the Chr 15 loci and 2.1 and 1.7-fold greater than the effects conferred by the Chr 19 loci, respectively (Table 2). At approximately − 2.0, the negative ratio between the effects on seed protein and oil contents was also greater for the Chr 20 loci than the Chr 15 or 19 loci.

### Genome-wide association analyses for trait-specific QTL on seed protein and oil contents

With each major significant allele having a negative ratio between protein and oil contents of at least − 1.6, specific analysis to identify alleles which increase one trait while having a neutral or positive effect on the other was also carried out. Using MTMM, trait-specific QTL, which lacked pleiotropy, were identified that included 17 significant SNPs with trait-specific effects for protein and oil contents from four LD blocks distributed on Chrs 5 and 10 (Table 3). Six additional SNPs were identified at 25% suggestive levels on Chrs 1, 5, 9, 10 and 18 (Table S11).

**Table 3:**
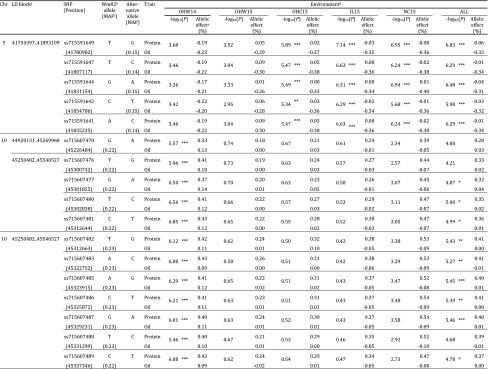
Trait-specific significant SNPs identified by multi-trait mixed model

*, suggestive threshold (25%); **, suggestive threshold (10%); ***, genome-wide significance threshold (5%)

^a^Linkage disequilibrium (LD) blocks were constructed based on four-gamete method. Blocks were merged, if adjacent blocks were separated by < 10 kb

^b^Williams 82

^c^Minor allele frequency

^d^OHW14, Wooster, OH, 2014; OHW15, Wooster, OH, 2015; OHC15, Columbus, OH, 2015; IL15, Urbana, IL, 2015; NC15, Plymouth, NC, 2015; ALL, all environments

^e^Allelic effect of alternative allele relative to Williams 82

**Fig. 4:**
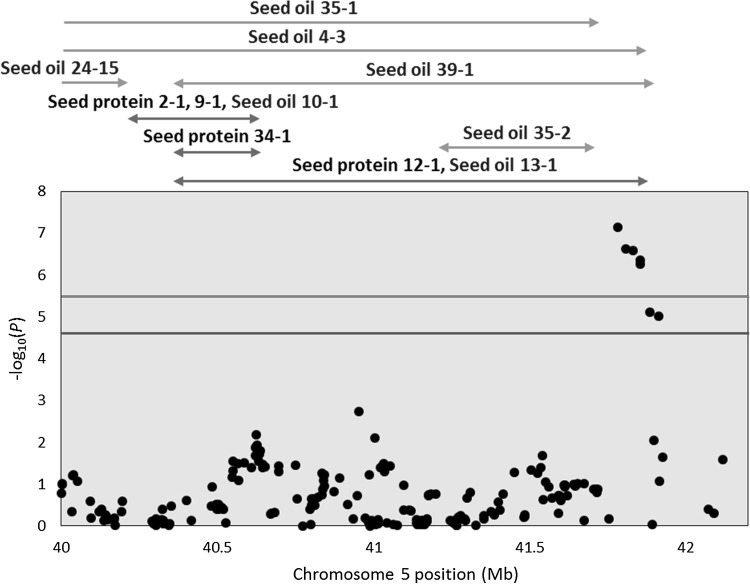
The 40–42.5 Mb region on Chr 5 covering significantly associated trait-specific SNPs identified by multi-trait mixed model. Negative log_10_*P*-values of for the Illinois 2015 environment (IL15) are plotted against physical genomic position (Glyma.Wm82.a2.v1). Horizontal lines are as described in Fig. 3. Previously identified QTL are indicated with horizontal arrows and were obtained from SoyBase (http://soybase.org)

On Chr 5, five SNPs (ss715591649, ss715591647, ss715591644, ss715591642 and ss715591641) within the single LD block were identified as significant in OHC15, IL15, NC15 and ALL (Table 3). These five SNPs were positioned within 132 kb at the distal end of Chr 5, and many previously known QTL for protein and oil contents have been mapped to this genomic region (Fig. 4). The allelic effects, based on the alternative allele relative to Williams 82, were near zero for protein content in all environments, except OHW14. The alternative allelic effect for oil was negative (average value of − 0.35%), regardless of environment.

**Fig. 5:**
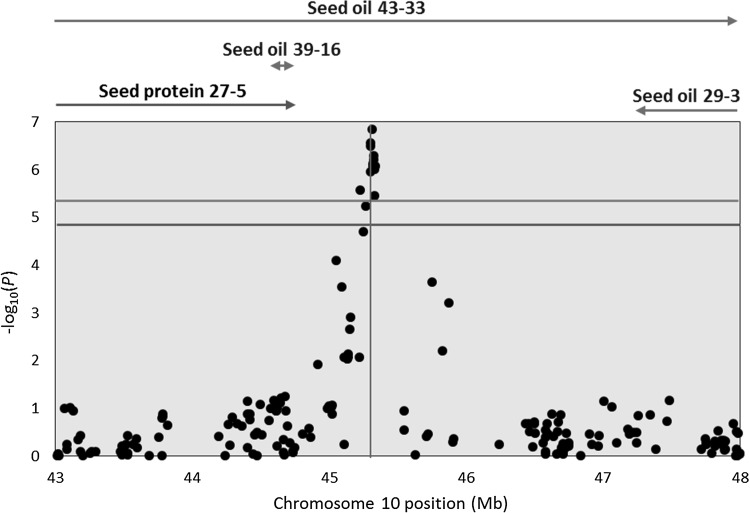
The 43–48 Mb region on Chr 10 covering significantly associated trait-specific SNPs identified by multi-trait mixed model. Negative log_10_*P*-values of for the Wooster, Ohio 2014 environment (OHW14) are plotted against physical position (Glyma.Wm82.a2.v1). Horizontal lines and arrows are as described in Fig. 4. The maturity gene (*E*2) indicated by the vertical line is coincident with these significant markers

The significant trait-specific markers on Chr 10 were within three LD blocks, with all SNPs significant only in OHW14 or ALL (Table 3). The SNPs co-located with a single previously identified QTL for oil content (seed oil 43–33, Mao et al. 2013) (Fig. 5). The effect of the alternative allele for the significant SNPs within each LD block was positive for both protein and oil contents in OHW14 (0.33 to 0.43% and 0.09 to 0.14%, respectively). However, for the remaining four individual environments and ALL, the effect was positive for protein content (0.18 to 0.54%) and nearly neutral for oil content (-0.10% to 0.10%). These significantly associated markers were coincident with the maturity gene *E2*/*GmGIa* (Watanabe et al. 2011; Fig. 5).

Since trait-specific effect QTL on Chr 10 were co-localized with the maturity gene, association analyses were implemented with maturity data available from two environments (OHW14 and NC15) (Figs. S11, S12). A total of seven LD blocks with markers at the significant or suggestive SNPs (5% to 25% levels) were identified on four chromosomes (Chrs 6, 8, 10 and 11) (Table S12). One marker on Chr 6 and two markers on Chr 10 had significance at the genome-wide level. While the Chr 6 marker (ss715593866) did not co-localize with any QTL for protein and oil contents (Table S12), the two markers on Chr 10 (ss715607470 and ss715607481) co-located with trait-specific effect QTL for protein and oil contents in OHW14 and were coincident with the maturity gene *E2*/*GmGIa* (Fig. S13).

### Genome-wide association analyses for amino acid contents

**Fig. 6:**
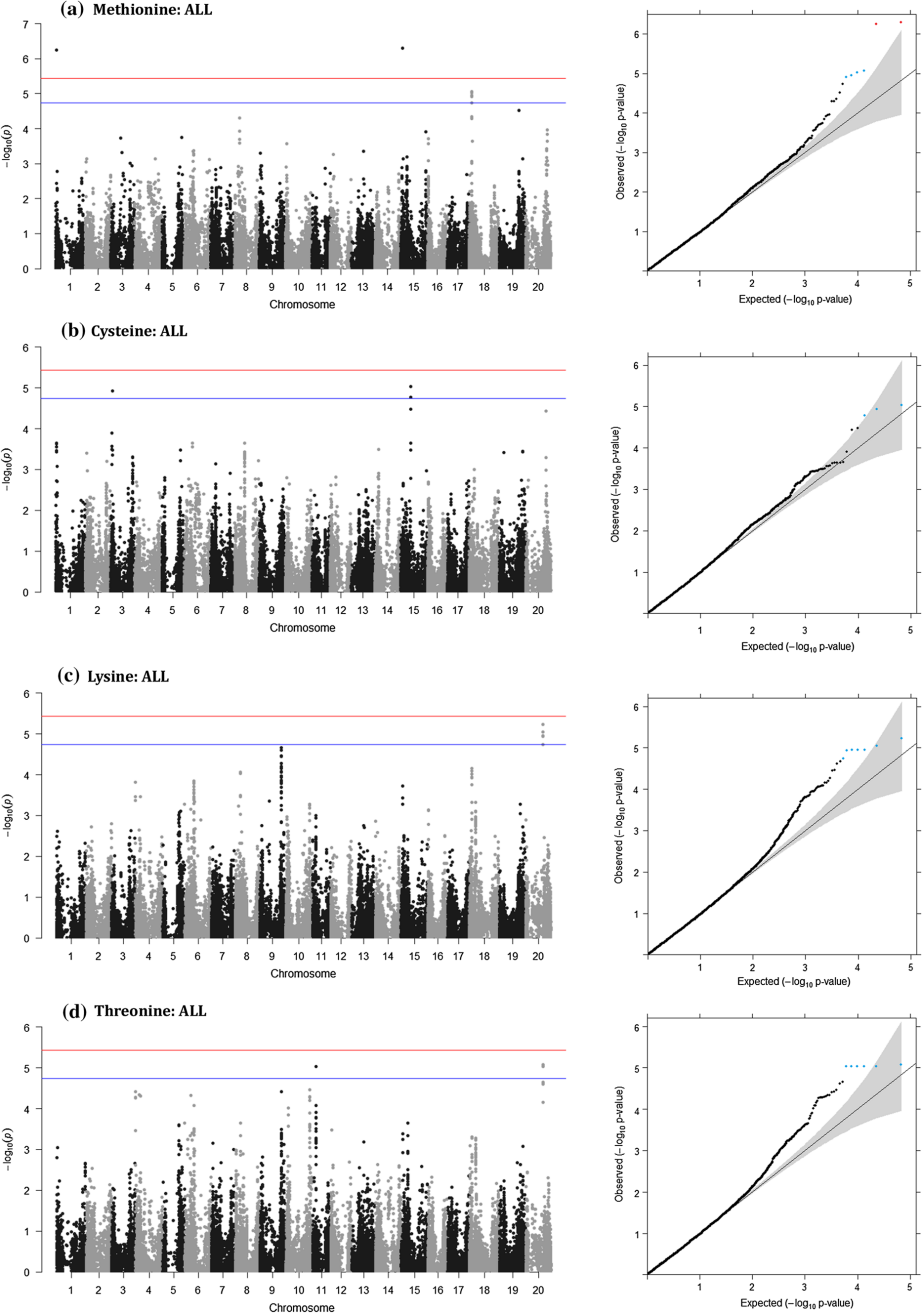
Manhattan plots (left) and QQ-plots (right) for genome-wide association study of the 621 soybean accessions using multi-locus mixed model for methionine (**a**), cysteine (**b**), lysine (**c**) and threonine (**d**) on a g kg cp^−1^ basis across all environments (ALL). Horizontal lines, markers and shading are as described in Fig. 3

Association analyses were also performed for the seed content of four amino acids, Met, Cys, Lys and Thr (g kg^−1^ cp), by each environment (OHW14, OHW15, OHC15, IL15 and NC15) and all environments combined (ALL) with both CMLM (Figs. S14, S15, S16, S17) and MLMM models (Figs. 6, S18, S19, S20, S21). The qq-plots of both CMLM (Figs. S14, S15, S16, S17) and MLMM models (Figs. 6, S18, S19, S20, S21) showed a sharp deviation from the expected *P* value distribution at the tail, indicating that false-positive and false-negative associations were effectively controlled. Using the same genome-wide significance threshold as applied for the protein and oil GWAS by MLMM, three, one, one and four SNPs were identified as significantly associated to Met, Cys, Lys and Thr, respectively, for a non-redundant set of eight SNPs (Table 4).

**Table 4:** Linkage disequilibrium (LD) blocks significantly associated with amino acid content (g kg^−1^ crude protein) by multi-locus mixed model

Chr	LD block position range^a^	Most significant SNP/LD block	Position	Trait environment	− log_10_(*P*)
1	1237296–1314722	ss715578474	1309778	Met-OHC15	6.86***
				Met-ALL	6.26***
				Met-IL15	5.37**
3	42759210–42819489	ss715586331	42783646	Cys-IL15	5.72***
9	41468783–41529869	ss715604076	41499208	Thr-OHC15	5.66***
10	45250482–45546527	ss715607486	45325872	Thr-NC15	6.40***
11	5865872–5987980	ss715610921	5886407	Thr-IL15	5.49***
				Thr-NC15	5.08*
				Thr-ALL	5.03*
15	3863922–3985288	ss715621799	3936757	Met-ALL	6.30***
				Met-OHC15	5.74***
				Met-NC15	4.78*
18	4886585–4996669	ss715631030	4953024	Met-OHC15	5.44***
				Met-ALL	4.74*
20	31554795–32384035	ss715637294	32282623	Thr-OHW14	10.88***
				Lys-OHW14	10.25***
				Thr-ALL	5.03*
				Lys-ALL	4.95*

*, suggestive threshold (25%); **, suggestive threshold (10%); ***, genome-wide significance threshold (5%)

^a^LD blocks were constructed based on four-gamete method. Blocks were merged, if adjacent blocks were separated by < 10 kb

Results from CMLM and MLMM models were similar for significant SNPs; thus, only results from MLMM will be discussed here. On 13 different chromosomes, a total of 35 LD blocks were represented by at least one SNP per block which was significantly or suggestively associated with the four amino acid contents, and similar to protein and oil contents, the significance of the association was often environmentally dependent (Figs. S18, S19, S20, S21, Table S13). Ten, seven, nine and five LD blocks were significantly or suggestively associated SNP markers for Met, Cys, Thr and Lys, respectively (Table S13). While many LD blocks were relatively near each other (less than 1 Mb), only two LD blocks (Gm15:3745323–3851525 and Gm20:31554795–32384035) had SNPs significantly or suggestively associated with more than one amino acid.

At the genome-wide significant threshold, a total of eight LD blocks had at least one significantly associated SNP. Significant LD blocks for Met resided on Chrs 1 (1237296–1314722 bp), 15 (3863922–3985288 bp) and 18 (4886585–4996669 bp) (Table 4). A single significant LD block for Cys resided on Chr 3 (42759210–42819489 bp). Significant LD blocks for Thr resided on Chrs 9 (41468783–41529869 bp), 10 (45250482–45546527 bp) and 11 (5865872–5987980 bp), while a fourth LD block on Chr 20 (31554795–32384035 bp) was significantly associated with both Thr and Lys (Table 4). The associations tended to be environmentally specific, with each of these loci associated in only one or two environments at a significant or suggestive level. Of interest, ss715621799 on Chr 15, which was identified by MLMM as significantly associated with protein and oil contents in OHC15 (Table 2), was the most significant SNP for Met in OHC15 and ALL. Similarly, the Thr-associated marker, ss715607486, on Chr 10, was significant for trait-specific effect QTL for protein and oil contents in OHW14 (Table 3) and ss715637294 on Chr 20, which is identified by MLMM for protein and oil contents in OHW14 (Table 2), was significant for both Thr and Lys in OHW14 only (Table 4).

## Discussion

Soybean is one of the major crop species that have extensive genotypic and phenotypic databases. Recent studies have taken advantage of the accumulated historical phenotypic data from USDA GRIN and the SoySNP50K data to investigate protein and oil contents using up to 12,000 soybean accessions (Bandillo et al. 2015; Hwang et al. 2014; Vaughn et al. 2014). Such studies are referential, because they utilized historically collected phenotype data, diagnosed genetic variation and suggested locally appropriate accessions (Jarquin et al. 2016). However, historical phenotypic data collected over time have important limitations, because the data often originated from various subsets of soybean accessions, which are frequently grown in un-replicated and incomplete blocks in different locations and years. Such variations in growing conditions among accessions can produce unreliable phenotypic data with relatively high residual values, especially important for quantitative traits sensitive to growing conditions (Specht et al. 2001). For example, soybean seed starch content was negatively influenced by the daily mean and minimum temperatures (Dhungana et al. 2017). Both starch and sucrose contents were negatively correlated with protein content but positively correlated with oil content (Li et al. 2012; Dhungana et al. 2017). Also, high levels of free amino acids were accumulated in soybean null mutant for storage proteins (Takahashi et al. 2003). Soybean protein and oil contents are significantly affected by growing conditions, such as day and night temperatures during the reproductive stages of growth (Gibson and Mullen 1996; Rotundo and Westgate 2009; Patil et al. 2017). Indeed, Hwang et al. (2014) reported that protein content of the 200 accessions measured in their study only moderately correlated with those reported in the USDA GRIN database (*r* = 0.61–0.62 and 0.77–0.78 for protein and oil contents, respectively). We also observed similar levels of correlation (0.62 and 0.80 for protein and oil contents, respectively) between USDA GRIN data and our own phenotypic data (scaled BLUP values) obtained from multi-environment tests. In the present study, we measured protein, oil and amino acid contents of 621 soybean accessions grown over five different environments in the Midwest and Southern USA in randomized and replicated plots and used those phenotypic data for QTL discovery. The data collected from replicated multi-location trials are inherently more reliable than that of un-replicated, incomplete tests. Thus, the GWAS results from the present study are expected to be more reliable than those, which have utilized seed protein and oil data from only the USDA GRIN database.

### Refinement of major protein and oil QTL on chromosomes 15 and 20

Many QTL for protein and oil contents have been mapped in a number of soybean populations (Van and McHale 2017). In particular, the genomic regions of Chrs 15 and 20 have been intensively studied (Chung et al. 2003; Diers et al. 1992; Kim et al. 2016; Nichols et al. 2006; Sebolt et al. 2000; Shibata et al. 2008; Tajuddin et al. 2003). Our MLMM analysis re-identified QTL for protein and oil contents at these known loci. In addition to specific genomic locations, our study also was in agreement with previous studies on the relative effects of these loci (Kim et al. 2016). The estimated allelic effects of the alternative allele (relative to Williams 82) were positive for protein content and negative for oil content, for both Chrs 15 and 20, with the relative effects of the Chr 20 QTL being larger than the Chr 15 QTL for both seed protein and oil contents (Table 2), and these results are similar to previous findings (Bandillo et al. 2015).

All four markers on Chr 15 were positioned within a 139 kb interval, representing a narrower region internal to the 535 kb interval defined by the Kim et al. (2016) fine-mapping study of this locus. Furthermore, three SNPs (ss715621777, ss715621799 and ss715621801) within 91 kb coincided with the 118 kb confidence interval of the meta-QTL for seed protein and oil contents (mPO15-2) (Van and McHale 2017). Notable functional candidates within this QTL window are *Glyma.15g049100* (vinorine synthase), *Glyma.15g049200* (sugar efflux transporter for intercellular exchange), *Glyma.15g050100* (fructose-1, 6-bisphosphatase) and *Glyma.15g050600* (glutamate decarboxylase and related proteins). Based on their putative functions, these genes may be involved in carbon partitioning and regulation of protein and oil contents in soybean.

Similar to the significantly associated markers on Chr 15, all seven markers on Chr 20 within 839 kb were positioned in the previously known QTL for protein and oil contents defined as the 10.1 Mb interval (Kim et al. 2016), the confidence intervals of the seed oil meta-QTL, mO20-3 (6.5 Mb), and the seed protein and oil meta-QTL, mPO20-3 (6.1 Mb) (Van and McHale 2017) (Fig. S22). Interestingly, Vaughn et al. (2014) reported a 1-Mb genomic region highly associated with protein content using a MS-2000 population consisting of primarily MG V accessions. Yet, this association disappeared in their analysis with MG III and IV lines. However, our 839 kb region identified with MG I to IV accessions resides within the 1 MB genomic region identified by Vaughn et al. (2014). Thus, our analysis refined the physical position of the association and extended the MGs to which the association applied. Within this QTL window, *Glyma.20g086900* (aldehyde dehydrogenase-related) and *Glyma.20g088400* (oxidoreductase, 2-oxoglutarate-Fe(II) oxygenase family protein) are positional candidate genes with potential functions in metabolism.

In addition to the markers on Chrs 15 and 20, two SNPs were identified as significant markers for protein and oil contents on Chr 19 (Table 2). Markers ss715635864 and ss715635870 on Chr 19 were identified as significantly associated in the MTMM-opposite analysis for protein and oil contents (negative pleiotropy). Both SNPs are positioned within seed protein QTL 16-2 (Chapman et al. 2003). Thus, ss715635765 and ss715635870 were implicated in both seed protein and oil contents in this study, where previously the association had been limited to seed oil content.

### Trait-specific QTL on chromosomes 5 and 10 break the negative correlation between seed protein and oil contents

While MLMM and MTMM-opposite effect approaches were used for identifying loci for protein and oil contents separately, the MTMM trait-specific approach provided the possibility of identifying loci, which break the negative relationship between seed protein and oil contents. The vast majority of QTL analyses for seed protein and oil contents have focused on these traits individually. However, using a traditional mixed linear model, a single instance of a positive relationship between protein and oil contents was reported from a locus on Chr 9 (ss715605091) (Hwang et al. 2014). In the present study, significant trait-specific QTL for oil or protein contents were identified on Chrs 5 and 10, respectively (Table 3).

Five trait-specific effect SNPs on Chr 5 were highly significant under multiple environments, with allelic effects that were generally negligible (positive or negative) on protein content and relatively large positive effects on oil content attributed to the major allele (Williams 82) (Table 3). Interestingly, all 12 significantly associated trait-specific effect SNPs on Chr 10 were significant only in OHW14 and ALL, with the positive effects increasing protein content and provided from the non-Williams 82 allele, which, in this case, was the major allele (Table 3). These 12 significant trait-specific effect SNPs on Chr 10 coincided with *E2*/*GmGIa* (Watanabe et al. 2011).

Co-localizations of QTL for seed composition and maturity have been previously noted. Recently, Patil et al. (2018) identified QTL for protein, oil and sucrose contents, some of which were co-localized with the maturity loci *E1* and *E4*. Jun et al. (2008) also reported that 9 of 22 previously identified seed protein QTL were near or very close to QTL for maturity based on positioning of known maturity QTL from SoyBase (http://soybase.org) on their SSR genetic linkage map. The environment as well as the dates of first flower and maturity affects the temperature experienced during seed fill. The effect of temperature during seed fill on seed protein and oil contents has been previously reported, though the effects are inconsistent. Song et al. (2016) found that the seed contents of crude protein, water-soluble protein and protein plus oil were all positively correlated with an accumulated temperature ≥ 15 C and mean daily temperature. Wolf et al. (1982) also concluded that protein content was positively correlated with a higher temperature. Temperature effects on protein and oil contents have also been examined in a set of soybean cultivars with MG 00-VIII, in which oil content increased with increasing temperature up to a maximum at a mean temperature of 28 C and protein plus oil showed a positive correlation with temperature (Piper and Boote 1999; Thomas et al. 2003). Though population structure and differing temperatures during seed fill were confounded, correlation of maturity with seed protein and oil contents that presumably result from a temperature effect on grain fill have been summarized in Patil et al. (2017). Patil et al. (2017) report that late maturity group soybean lines (MG V-X) tended to have higher protein content and lower oil content than early maturity group lines (MG 000-II). In our study, the same correlations between plant maturities and the seed protein and oil contents were observed within MG I-IV. MG IV soybean lines showed highest protein content (37.8%) and lowest oil contents (16.14%), whereas MG I lines had lowest protein content (35.86%) and highest oil content (18.82%). Indeed, breeders may find that the environmental specificity and association with maturity of the Chr 10 locus and the high frequency of the positive alleles for the Chr 10 as well as Chr 5 loci may limit their use in breeding programs.

### Identification of novel genomic regions for essential amino acids

Generally, soybean improvement for seed composition has focused on seed protein and oil contents. Additional research is needed for improvement of amino acid contents to changes the amino acid profile to meet a better nutrient profile of the soybean meal. Soybean seed storage proteins have relatively low sulfur-containing amino acids, Met and Cys as well as Lys and Thr (Patil et al. 2017; Warrington et al. 2015). Using GWAS, we were able to identify 8 novel genomic regions associated with Met, Cys, Lys and Thr (g kg^−1^ cp), though the effects of each QTL for the four amino acids contents were not stable across environments (Table 4), consistent with previous studies on the amino acid composition of soybean seed (McClure et al. 2017). Interestingly, both ss715621799 on Chr 15 and ss715637294 on Chr 20 were also identified as QTL for protein content under the same environments (Table 2). Similarly, ss715607486 on Chr 10 was identified not only as significantly associated with Thr content (g kg^−1^ cp), but also as trait-specific effect QTL for protein content (Table 3). If associated SNPs are expanded to those identified at the suggestive level (Table S11), there is some commonality between previously identified loci: ss715589167 on Chr 4 (Met QTL, Vaughn et al. 2014), ss715593768 on Chr 6 (Met QTL, Warrington et al. 2015), ss715604045 on Chr 9 (Lys QTL, Warrington et al. 2015) and ss715631100 on Chr 18 (Met QTL, Panthee et al. 2006). Thus, although significance levels of these four SNPs were not above genome-wide threshold, these four SNPs act to confirm results of previous studies.

### Geographic and MG distribution of key haplotypes reveal their potential utility in breeding programs

**Fig. 7:**
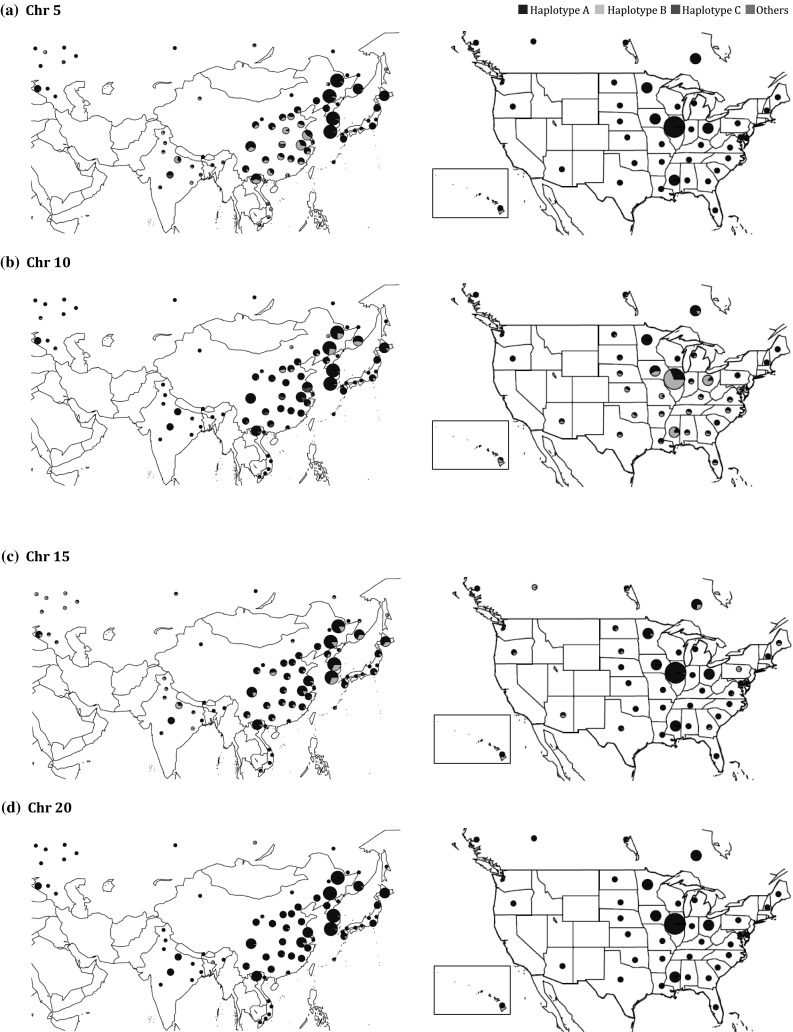
Distribution of haplotypes of trait-specific QTL for protein and oil on Chr 5 (**a**) and Chr 10 (**b**) and QTL for protein and oil on Chr 15 (**c**) and Chr 20 (**d**). The frequency of each haplotype, illustrated in pie charts, was placed according to the geographic locations of major populations from Russia, Asia and North America. Size of pie chart is correlated to the number of accessions in the region. Haplotypes are as described in Table 5. The figure map was created using the R package ‘maps’ and ‘mapdata’ in the R project

While potentially valuable alleles were identified by both the MLMM and MTMM the analyses, we were interested in exploring the distribution of these alleles across geographic regions and MGs in order to assess their potential utility in specific breeding programs. Thus, haplotype distributions of LD blocks containing the most significant marker for each chromosomal region (Chr 5: 41754397–41893109; Chr 10: 45250482–45546527; Chr 15: 3863922–3985288; Chr 20: 31554795–32384035), were surveyed within the GRIN database based on MG (Table 5) and geographic origin (Fig. 7). To determine the utility of key alleles in the US, we focused on MGs II and III, which are the primary MGs grown in the major US soybean producing states (Rinker et al. 2014).

**Table 5:**
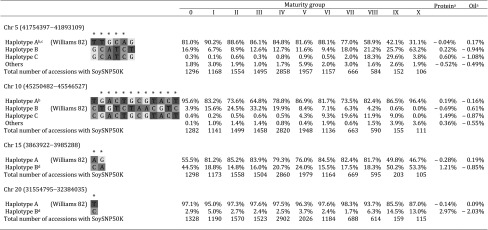
Haplotypes of QTL for protein and oil contents by maturity group on Chrs 5, 10, 15 and 20 (color table online)

Haplotypes were classified only with soybean accessions having SoySNP50K data (SoyBase, http://soyase.org.snps/) and maturity group information (GRIN, http://www.ars-grin.gov/cgi-bin/npgs/html/crop.pl?51)

*Significant SNPs at 5% as a genome-wide significance threshold

^a^Average seed protein or oil content (%) of individuals with this haplotype from the 621 Plant Introductions (PIs) minus the average of all 621 PIs for the ALL environment (average of 621 PIs: 37.60% Protein, 16.63% Oil at a 13% moisture basis)

^b^Haplotype contributing a positive effect as determined by MTMM analysis

^c^Haplotypes were classified only with soybean accessions having SoySNP50K data (SoyBase, http://soyase.org.snps/) and maturity group information (GRIN, http://www.ars-grin.gov/cgi-bin/npgs/html/crop.pl?51)

^d^Haplotype contributing a positive effect for seed protein content by MLMM analysis

For both Chrs 5 (41754397–41893109) and 10 (45250482–45546527), where the major haplotype allele (haplotype A) provided the positive effect for both protein and oil contents, haplotype A was prevalent across our targeted MGs (II & III) and geographic regions (US). The haplotype A on Chr 5 was found at 88.6% and 86.1% of the MG II and MG III germplasm in USDA Collection (Table 5), respectively, and was nearly fixed for all soybean producing states in the US (Fig. 7a), indicating a potential lack of utility in US soybean breeding programs. Haplotype A on Chr 10 was at a moderately lower frequency of 73.6% and 64.8% for the MG II and MG III germplasm in National Plant Germplasm System (http://www.ars-grin.gov/npgs/index.html), respectively (Table 5). Yet, haplotype A for Chr 10 was not the major allele for some regions across the US, including major soybean producing states, such as Illinois (31.3% allele frequency) (Fig. 7b). Thus, based on haplotype distribution alone, haplotype A of Chr 10 QTL could be utilized for soybean improvement by substitution of the Williams 82 allele at the locus. However, the Chr 10 QTL’s association with maturity and exhibited environmental instability represent potentially substantial functional constraints to its utility in breeding programs.

The distribution of haplotypes for Chrs 15 (3863922–3985288) and 20 (31554795–32384035) revealed a different story (Table 5). The minor allele, haplotype B for both Chrs 15 and 20, provided the positive effect on seed protein content and negative effect on seed oil content. For the Chr 15 locus, 14.8% of the MG II and 16.0% of the MG III accessions from USDA Collection contained haplotype B. In contrast, more than half of MGs IX and X germplasms contained haplotype B. While haplotype B from Chr 15 has a greater frequency in Asia and Russia, it is present at very low frequencies in many regions of the US and Canada (Fig. 7c). Thus, based on the haplotype distribution within the USDA Soybean Germplasm Collection, the potential utility of haplotype B of Chr 15 varies across MGs, but is high for MGs II and III and throughout the US.

Haplotype B of Chr 20 (31554795–32384035) was the minor allele across all MGs, with only 2.7% of the MG II and 2.4% of the MG III germplasm possessing this allele (Table 5). Similarly, across nearly all geographic regions, haplotype B is present at a very low frequency (< 5%) (Fig. 7d). While this locus has been studied for decades (Diers et al. 1992), it has just begun to be introgressed into modern US cultivars (Mian et al. 2017). Since its presence in the USDA Collection is very low across subpopulations, there is a high potential utility for this allele to be used in the development of high protein soybean cultivars for most regions of the US.

This GWAS study not only re-identified and narrowed down the windows of previously reported major QTL for protein and oil contents, but also found trait-specific effect on loci for both traits, indicating that it may be possible to reduce the negative relationship between protein and oil contents. While allelic distributions within the USDA Soybean Germplasm Collection may not be precisely representative of modern cultivars, the allelic distributions across MGs and geographic regions are informative and potentially useful in breeding for improvement of soybean protein and oil contents. Based on haplotype distributions for the key loci on Chrs 5, 10, 15 and 20, we suggest that haplotype B of Chrs 15 and 20 and potentially haplotype A of Chr 10 may be useful for improvement of seed protein and oil contents of North American soybean in many regions across the US.

#### Author contribution statement

SL and KV conducted genetic and field experiments, analyzed data and drafted/edited manuscript. MS and JL conducted field experiments and edited manuscript. RN contributed to selecting soybean accessions, conducted field experiments and edited manuscript. LKM and MARM designed and organized the project and edited manuscript.

## Electronic supplementary material

Below is the link to the electronic supplementary material.

Supplementary material 1 (XLSX 302 kb)

Supplementary material 2 (PDF 1793 kb)
