# Supplementary material for: Genome-wide association study of seed protein, oil and amino acid contents in soybean from maturity groups I to IV
Source: Theor Appl Genet. 2019 Feb 26;132(6):1639–59. doi: 10.1007/s00122-019-03304-5 (PMC6531425; doi:10.1007/s00122-019-03304-5)
Supplement: Supplementary file 2 — Supplementary material 2 (PDF 1793 kb) [file 122_2019_3304_MOESM2_ESM.pdf]

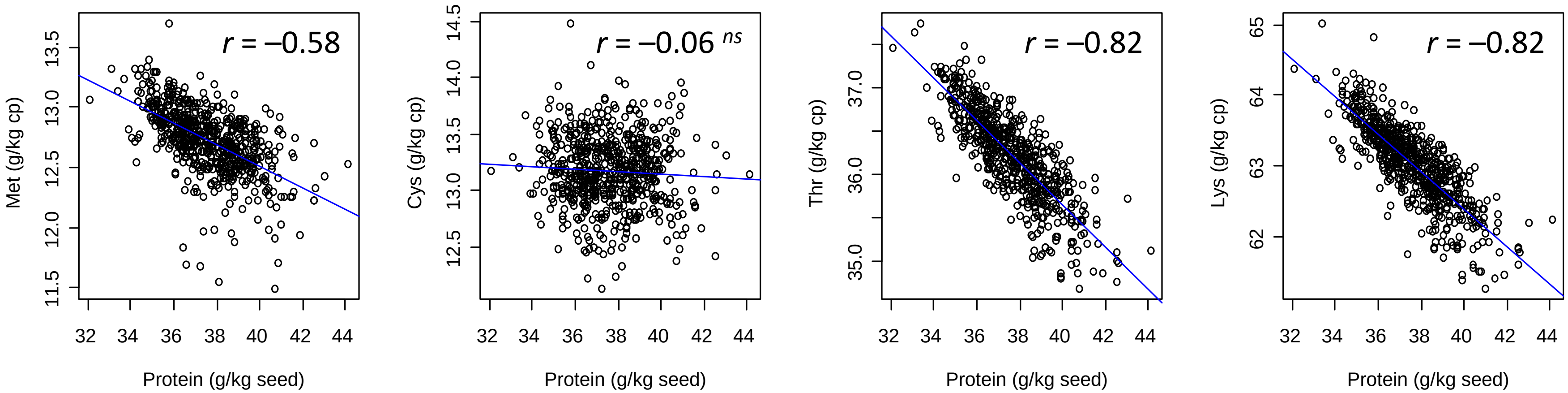

Fig. S1 Correlations between protein and amino acids by scaled BLUP values across all environments among the 621 plant introductions (P < 0.0001 for Met, Thr and Lys).

(a)

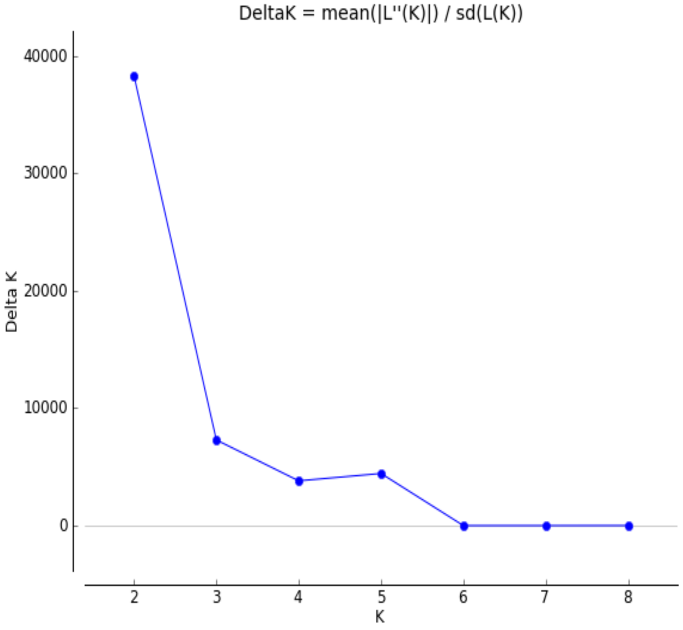

(b)

K	Reps	Mean LnP(K)	Stdev LnP(K)	Ln'(K)	|Ln''(K)|	Delta K
1	10	-2300952.59	1.4185	NA	NA	NA
2	10	-2118115.47	1.9477	182837.12	74600.19	38302.14266
3	10	-2009878.54	3.8661	108236.93	28196.35	7293.135146
4	10	-1929837.96	5.243	80040.58	19981.64	3811.088434
5	10	-1869779.02	5.5108	60058.94	24459.23111	4438.451997
6	10	-1834179.311	5593.685	35599.70889	88.057778	0.015742
7	10	-1798667.66	7191.4043	35511.65111	9544.48	1.327207
8	10	-1772700.489	1628.0735	25967.17111	6275.772222	3.854723
9	10	-1753009.09	3724.2806	19691.39889	NA	NA

Fig. S2 Results of *Delta K* analysis that determined the most like number of sub-populations from the STRUCTURE output

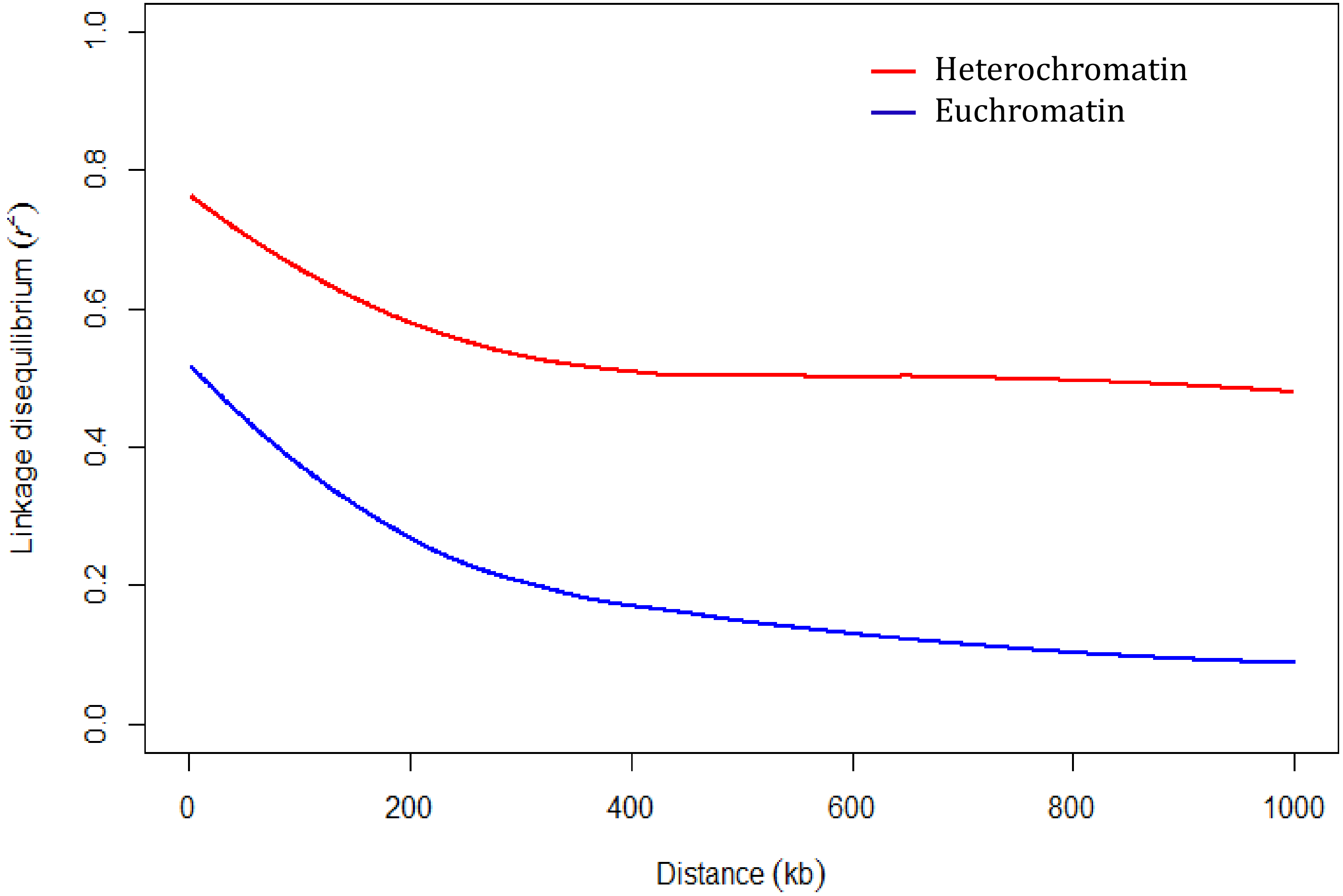

Fig. S3 Linkage disequilibrium (r^2) as a function of physical distance (kb) between marker pairs in heterochromatic and euchromatic chromosome regions.

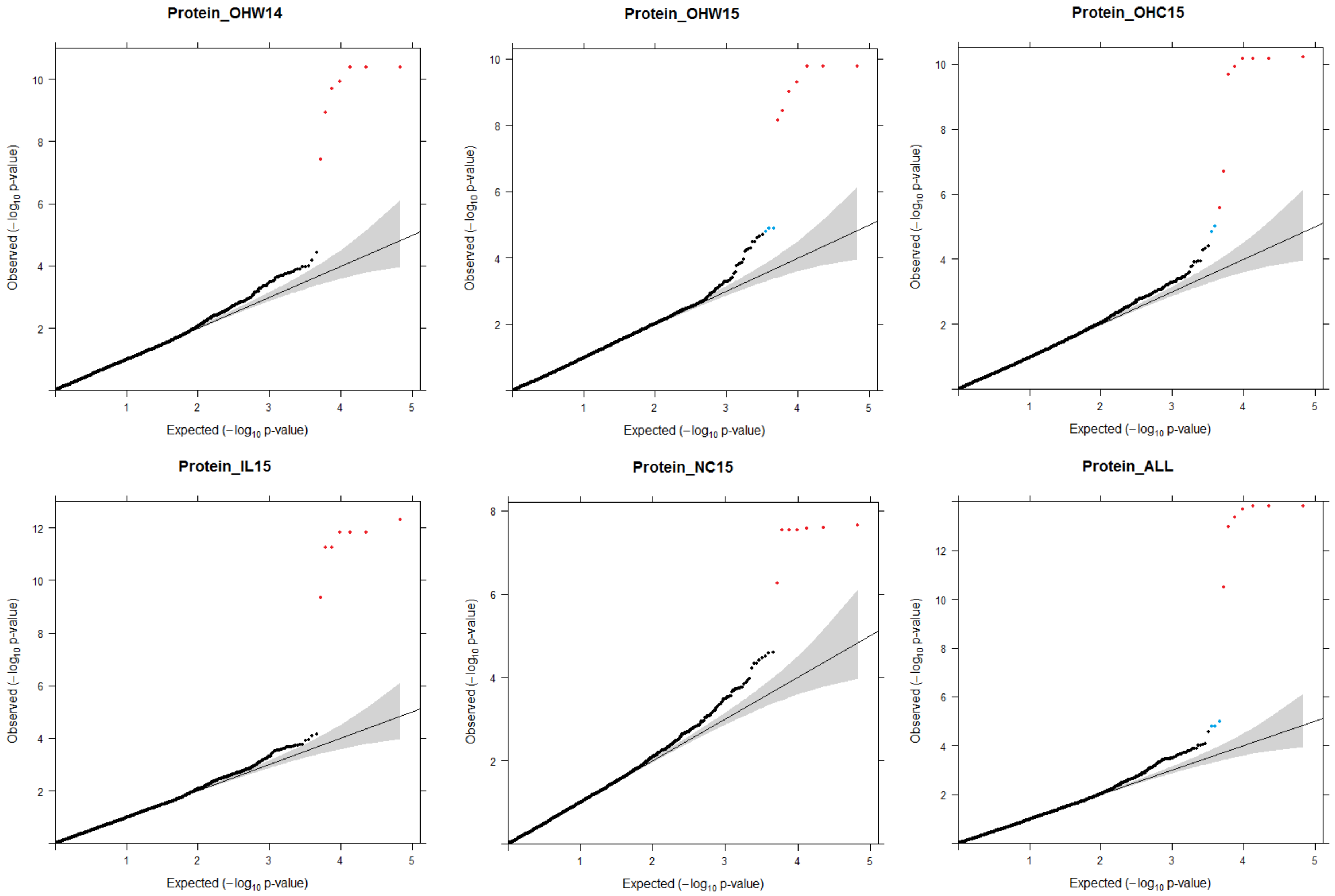

Fig. S4 QQ-plots for GWAS of 621 soybean accessions for seed protein content using CMLM. 34,014 SNPs were plotted for each environment and all environments combined. Red and blue dots represent the SNPs significantly associated at genome-wide significant threshold (5%) and suggestive significance thresholds (25%), respectively. Shaded region of the QQ-plots represent a 95% confidence interval.

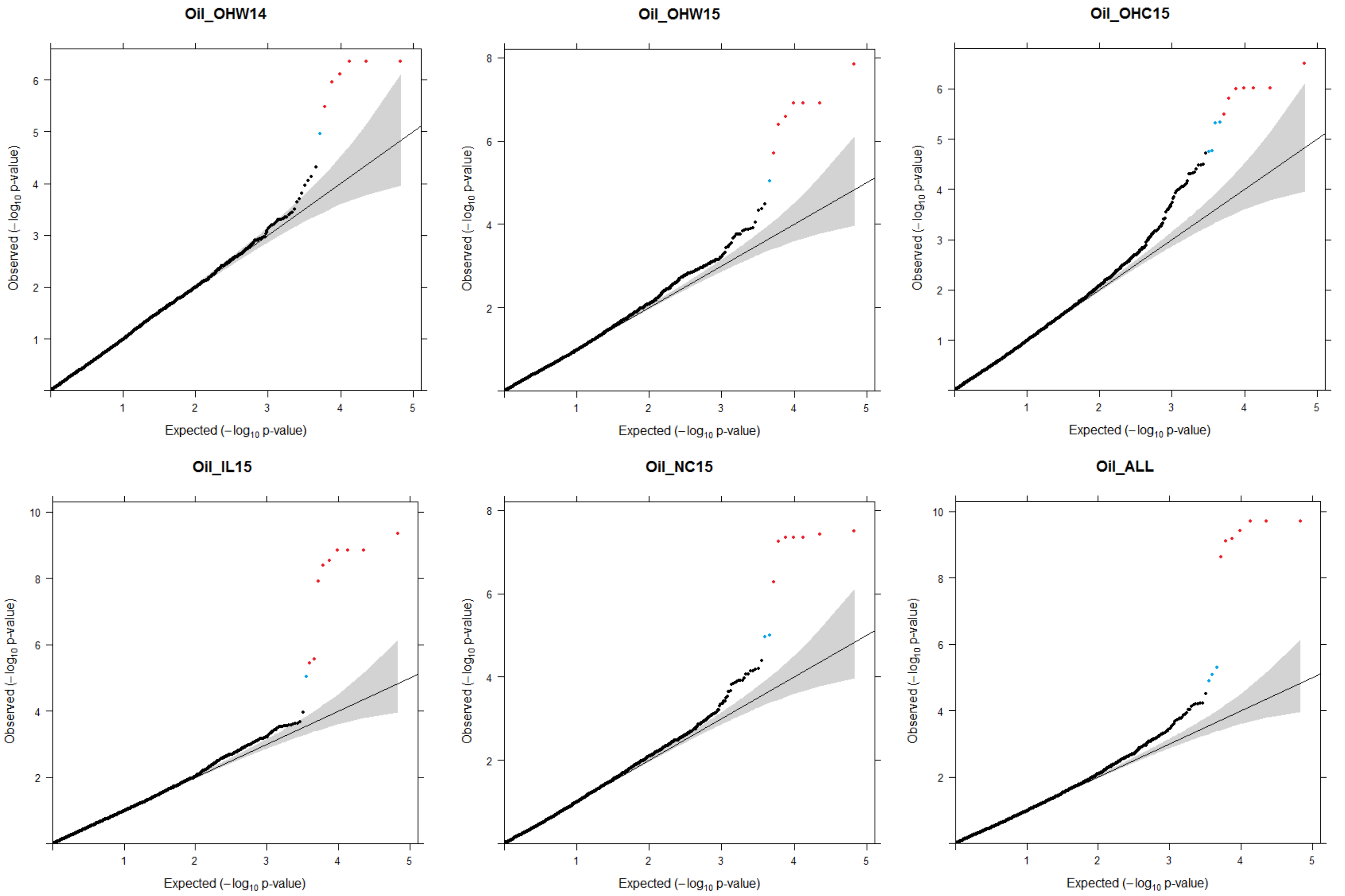

Fig. S5 QQ-plots for GWAS of 621 soybean accessions for seed oil content using CMLM. 34,014 SNPs were plotted for each environment and all environments combined. Red and blue dots represent the SNPs significantly associated at genome-wide significant threshold (5%) and suggestive significance thresholds (25%), respectively. Shaded region of the QQ-plots represent a 95% confidence interval.

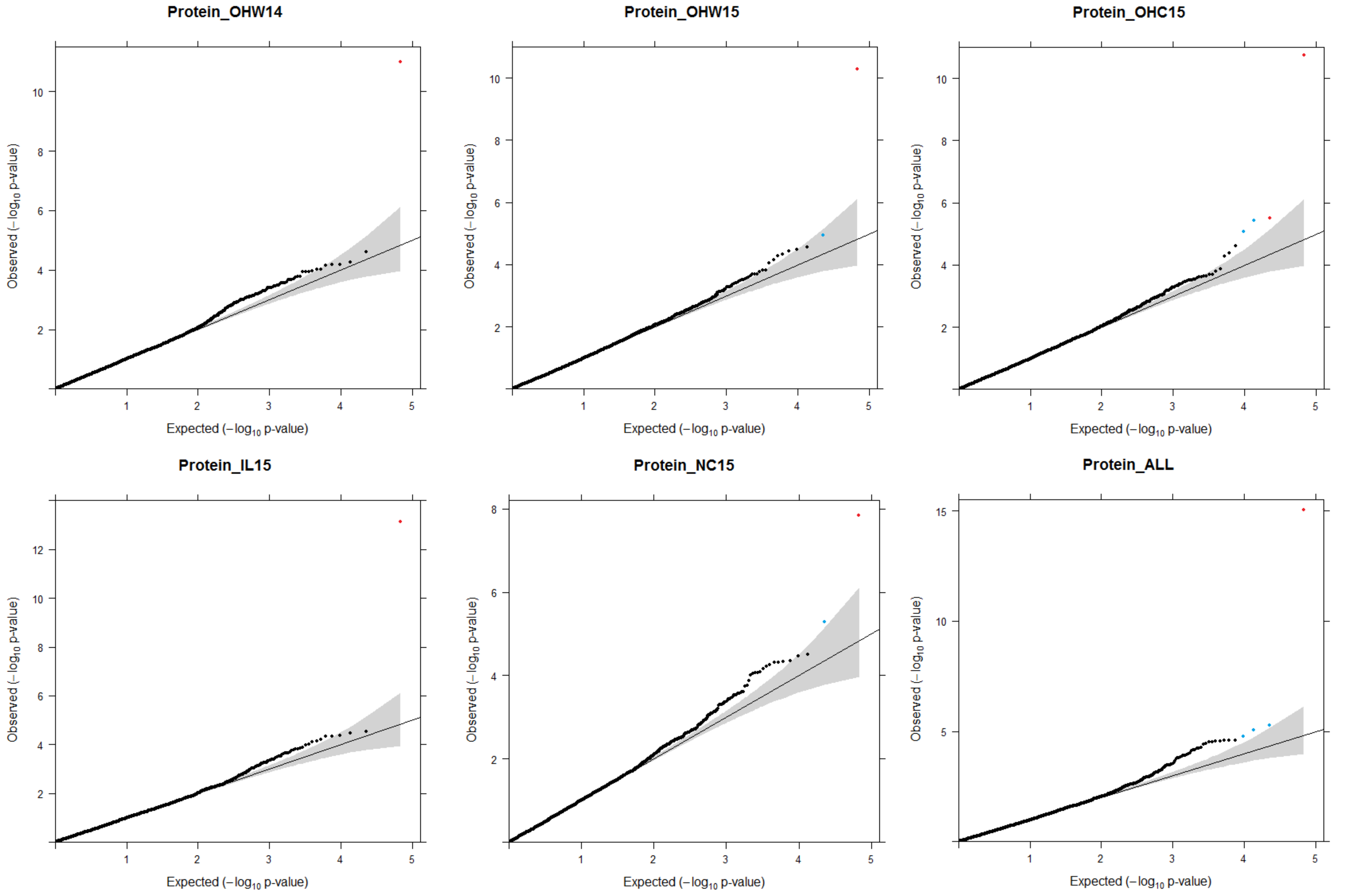

Fig. S6 QQ-plots for GWAS of 621 soybean accessions for seed protein content using MLMM. 34,014 SNPs were plotted for each environment and all environments combined. Red and blue dots represent the SNPs significantly associated at genome-wide significant threshold (5%) and suggestive significance thresholds (25%), respectively. Shaded region of the QQ-plots represent a 95% confidence interval.

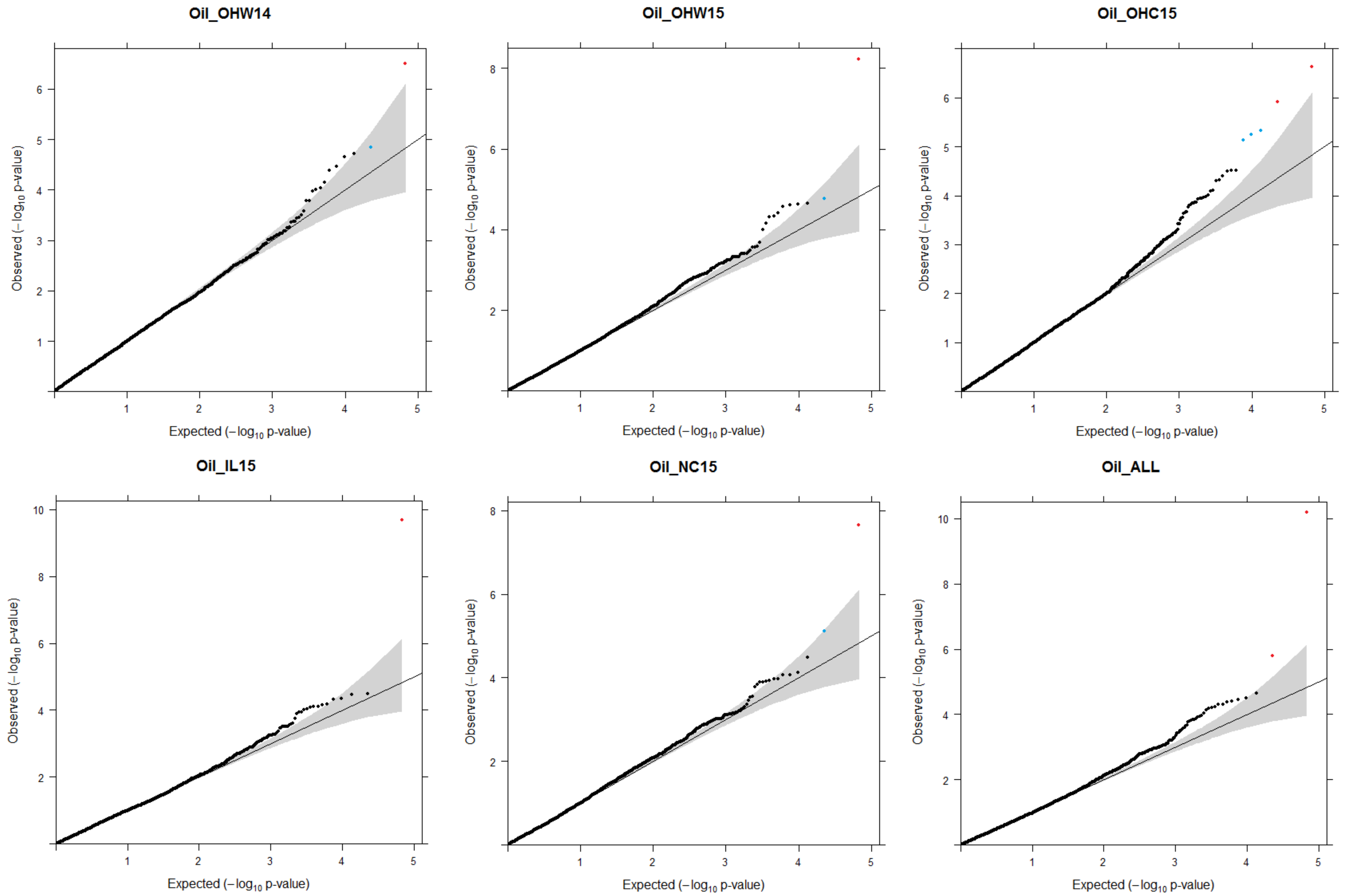

Fig. S7 QQ-plots (right) for GWAS of 621 soybean accessions for seed oil content using MLMM. 34,014 SNPs were plotted by each environment and all environments combined. Red and blue dots represent the SNPs significantly associated at genome-wide significant threshold (5%) and suggestive significance thresholds (25%), respectively. Shaded region of the QQ-plots represent a 95% confidence interval.

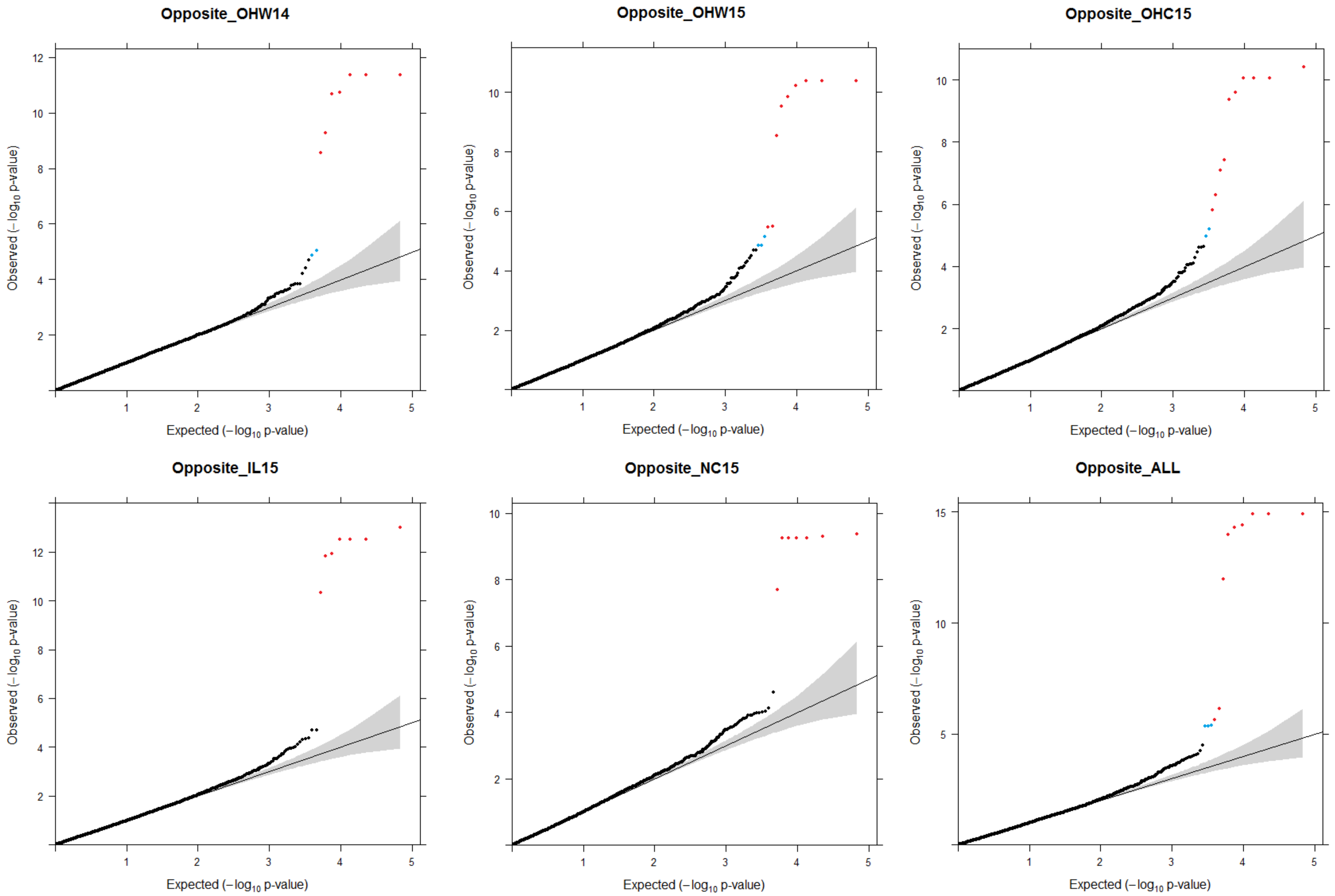

Fig. S8 QQ-plots for GWAS of 621 soybean accessions for seed protein and oil content using MTMM-opposite. 34,014 SNPs were plotted for each environment and all environments combined. Red and blue dots represent the SNPs significantly associated at genome-wide significant threshold (5%) and suggestive significance thresholds (25%), respectively. Shaded region of the QQ-plots represent a 95% confidence interval.

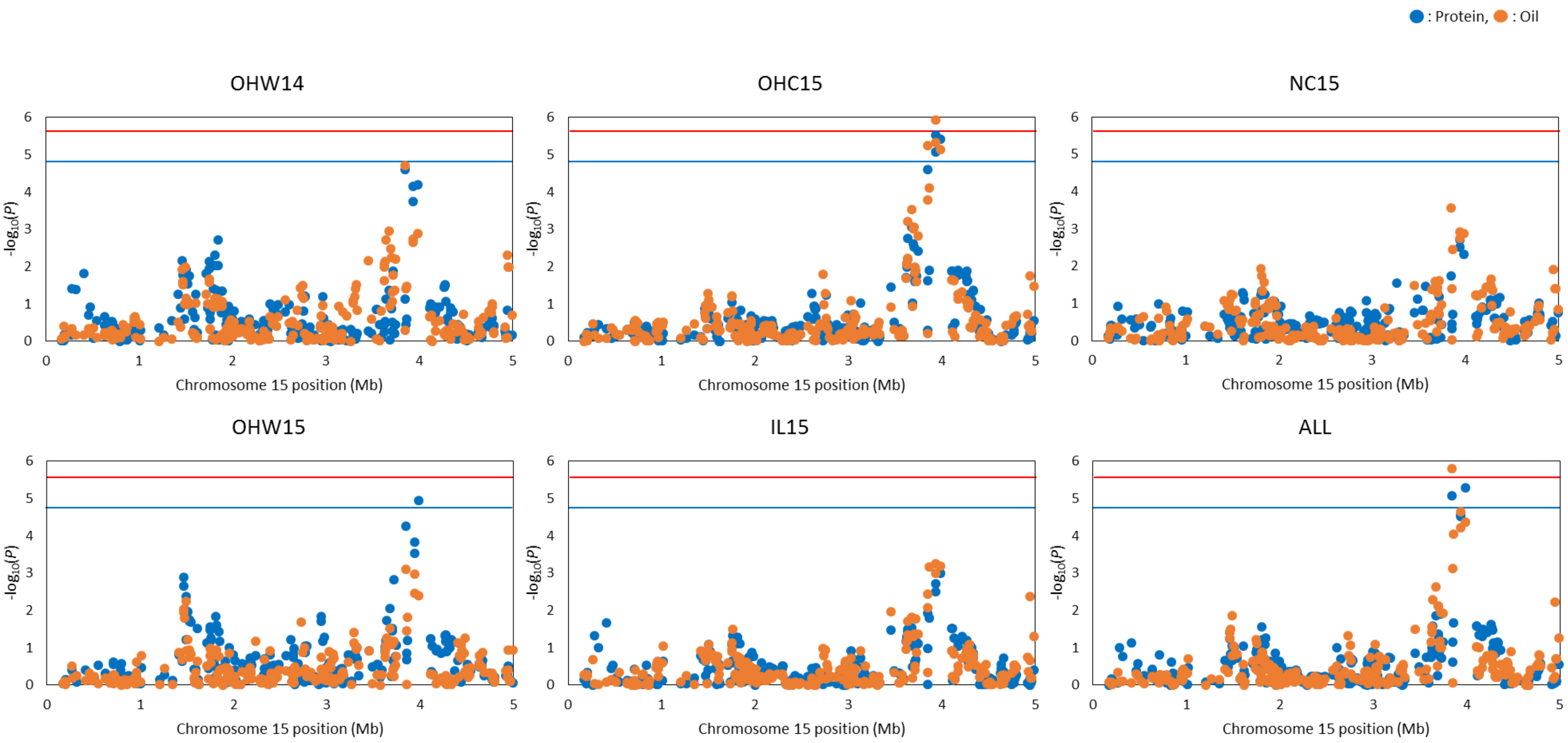

Fig. S9 Manhattan plots for protein and oil content by each environment for a 5-Mb region surrounding significantly associated SNPs on chromosome 15. Negative $\log_{10}$ *P*-values of MLMM are plotted against physical position (Glyma.W82.a2.v1). Red and blue horizontal lines represent genome-wide significant threshold (5%) and suggestive thresholds (25%), respectively.

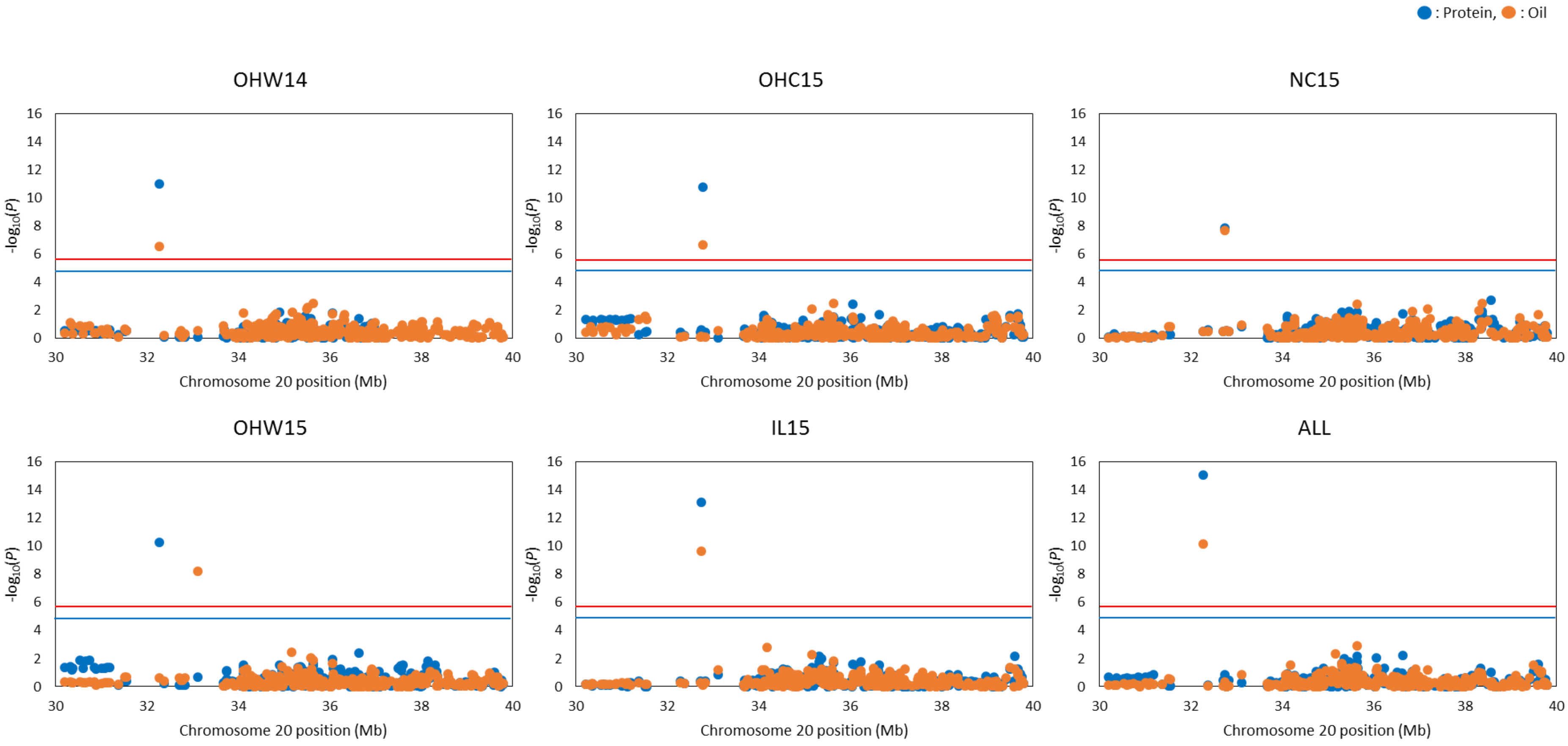

Fig. S10 Manhattan plots for protein and oil content by each environment for a 10-Mb region surrounding significantly associated markers on chromosome 20. Negative $\log_{10}$ *P*-values of MLMM are plotted against physical position (Glyma.W82.a2.v1). Red and blue horizontal lines represent genome-wide significant threshold (5%) and suggestive thresholds (25%), respectively.

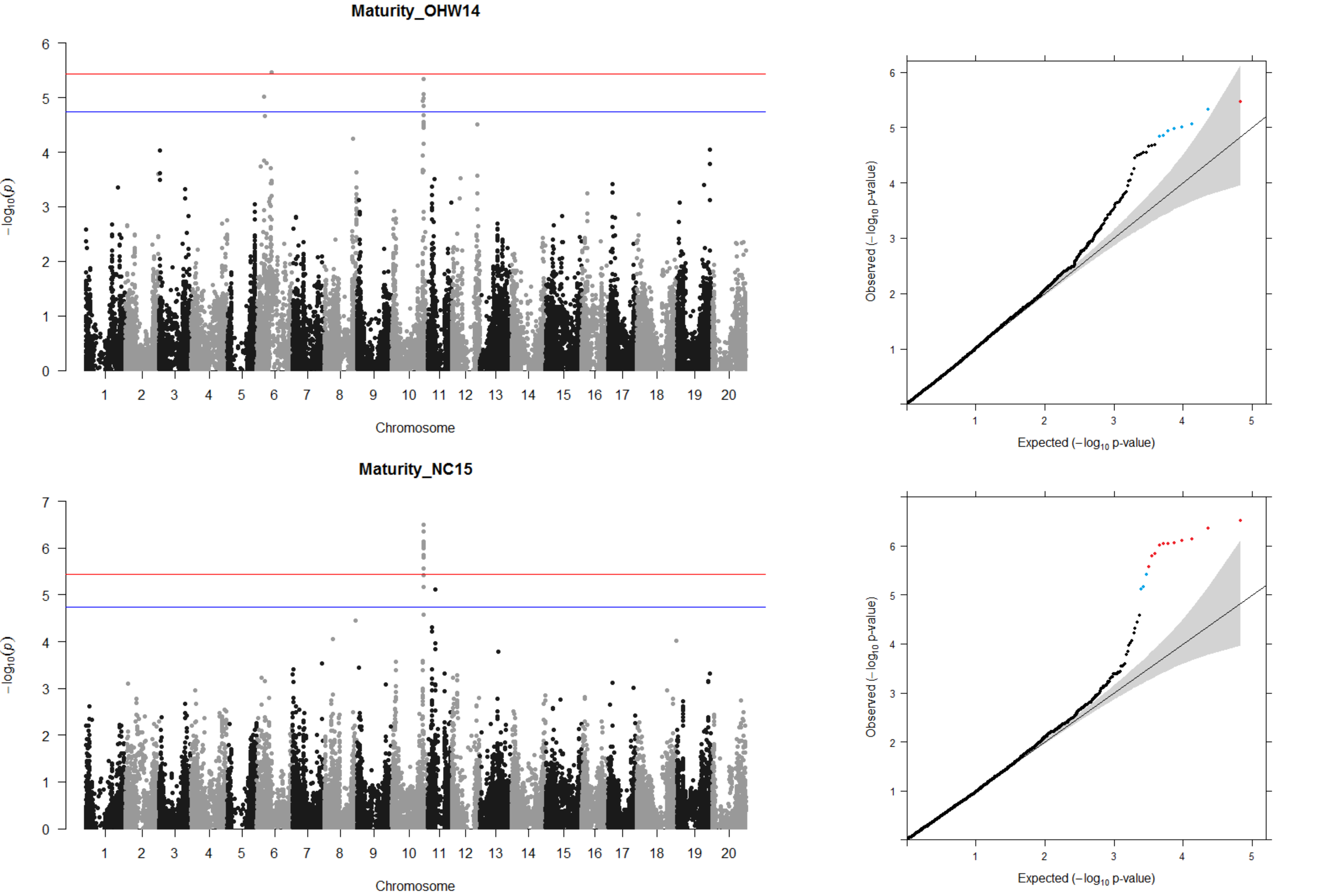

Fig. S11 Manhattan plots (left) and QQ-plots (right) for GWAS of 621 soybean accessions for maturity using CMLM. 34,014 SNPs were plotted by each environment and all environments combined. Red and blue horizontal lines in the Manhattan plots and markers in the QQ-plots represent the genome-wide significant threshold (5%) and suggestive significance thresholds (25%), respectively, and the SNPs significantly associated at those levels. Shaded region of the QQ-plots represent a 95% confidence interval.

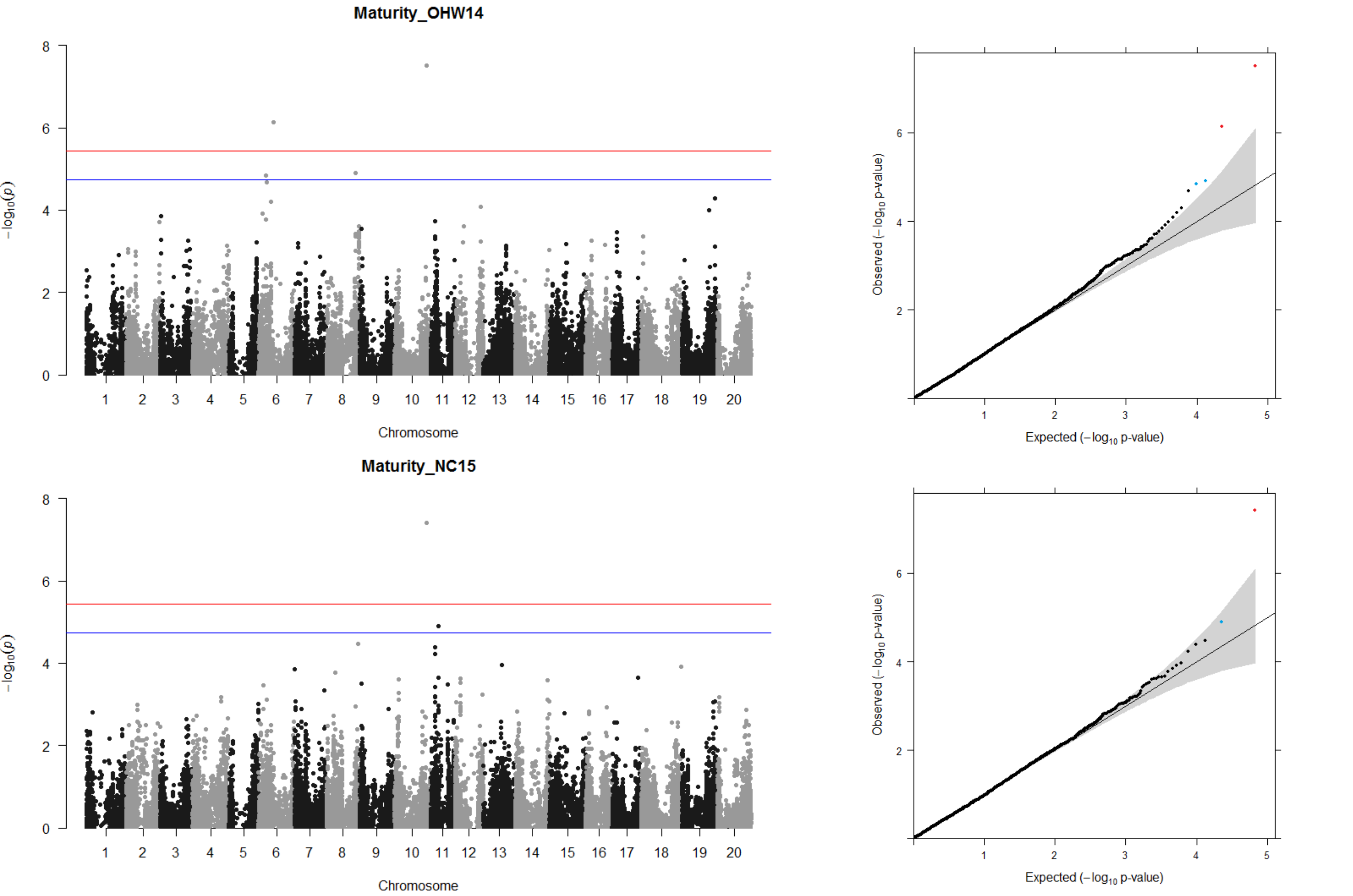

Fig. S12 Manhattan plots (left) and QQ-plots (right) for GWAS of 621 soybean accessions for maturity using MLMM. 34,014 SNPs were plotted by each environment and all environments combined. Red and blue horizontal lines in the Manhattan plots and markers in the QQ-plots represent the genome-wide significant threshold (5%) and suggestive significance thresholds (25%), respectively, and the SNPs significantly associated at those levels. Shaded region of the QQ-plots represent a 95% confidence interval.

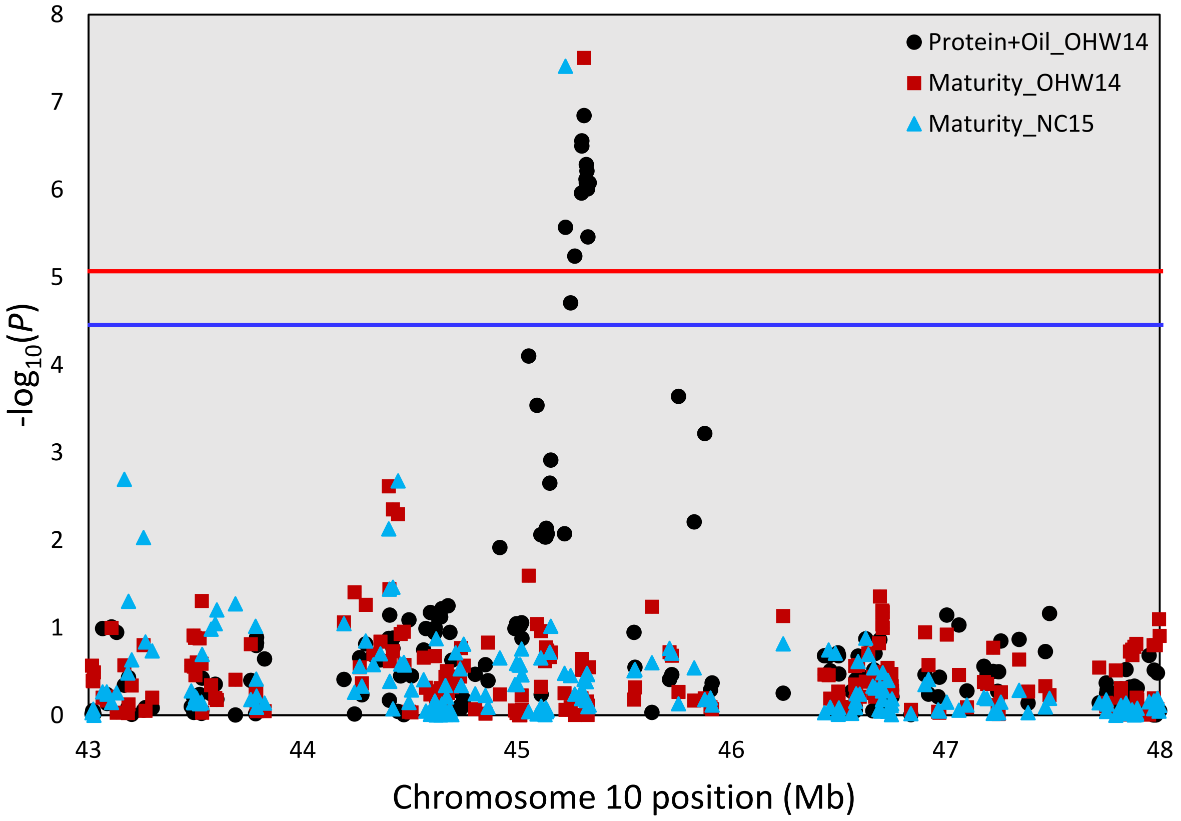

Fig. S13 Marker associations in the 43-48 Mb region surrounding significantly associated markers on Chr 10. Negative $\log_{10}$ *P*-values of MTMM for trait-specific protein and oil effects for OHW14 and MLMM for maturity in OHW14 and NC15 are plotted against physical position (Glyma.W82.a2.v1). Red and blue horizontal lines represent genome-wide (5%) and suggestive significance thresholds (25%), respectively.

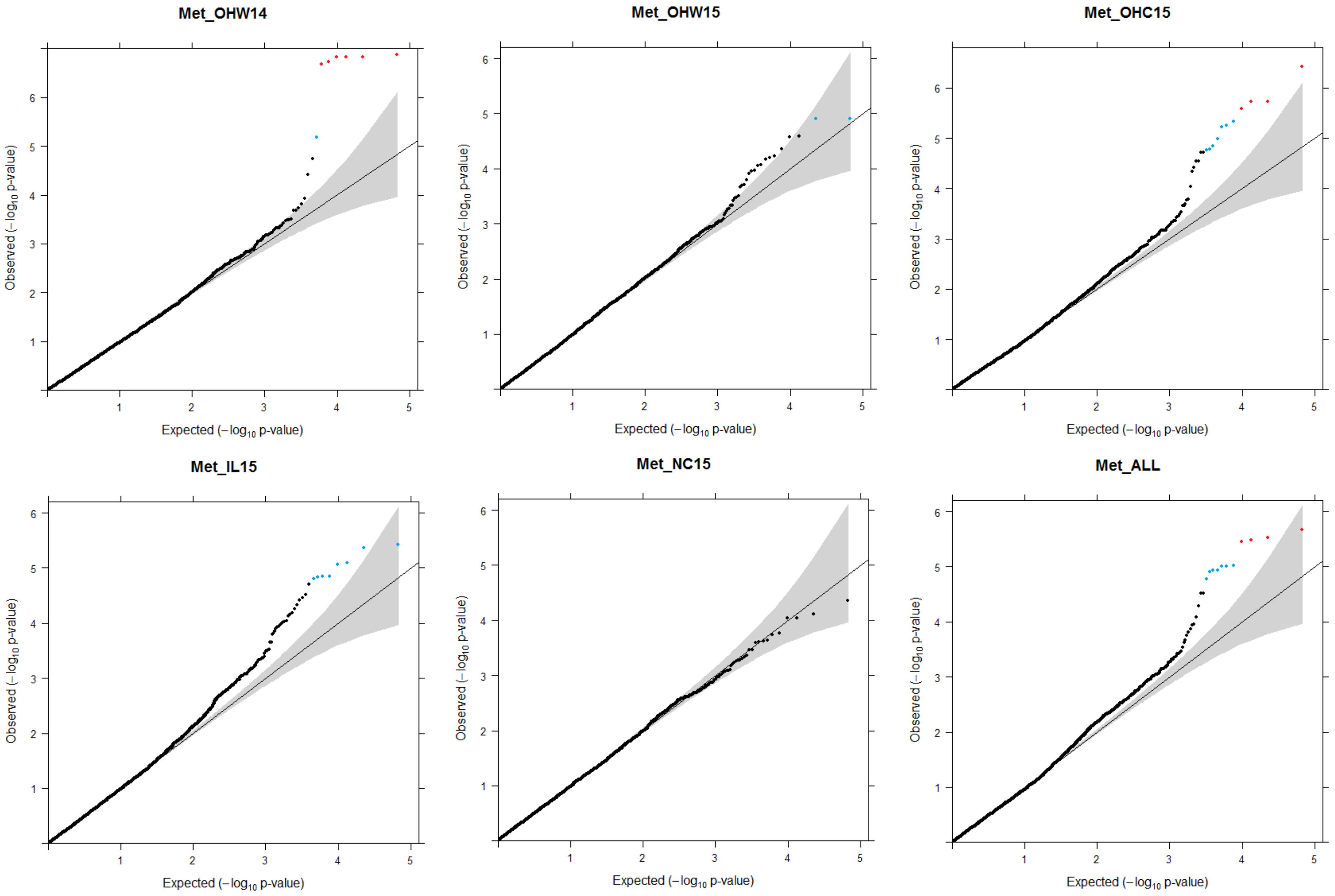

Fig. S14 QQ-plots (right) for GWAS of 621 soybean accessions for Met using CMLM. 34,014 SNPs were plotted by each environment and all environments combined. Red and blue dots represent the SNPs significantly associated at genome-wide significant threshold (5%) and suggestive significance thresholds (25%), respectively. Shaded region of the QQ-plots represent a 95% confidence interval.

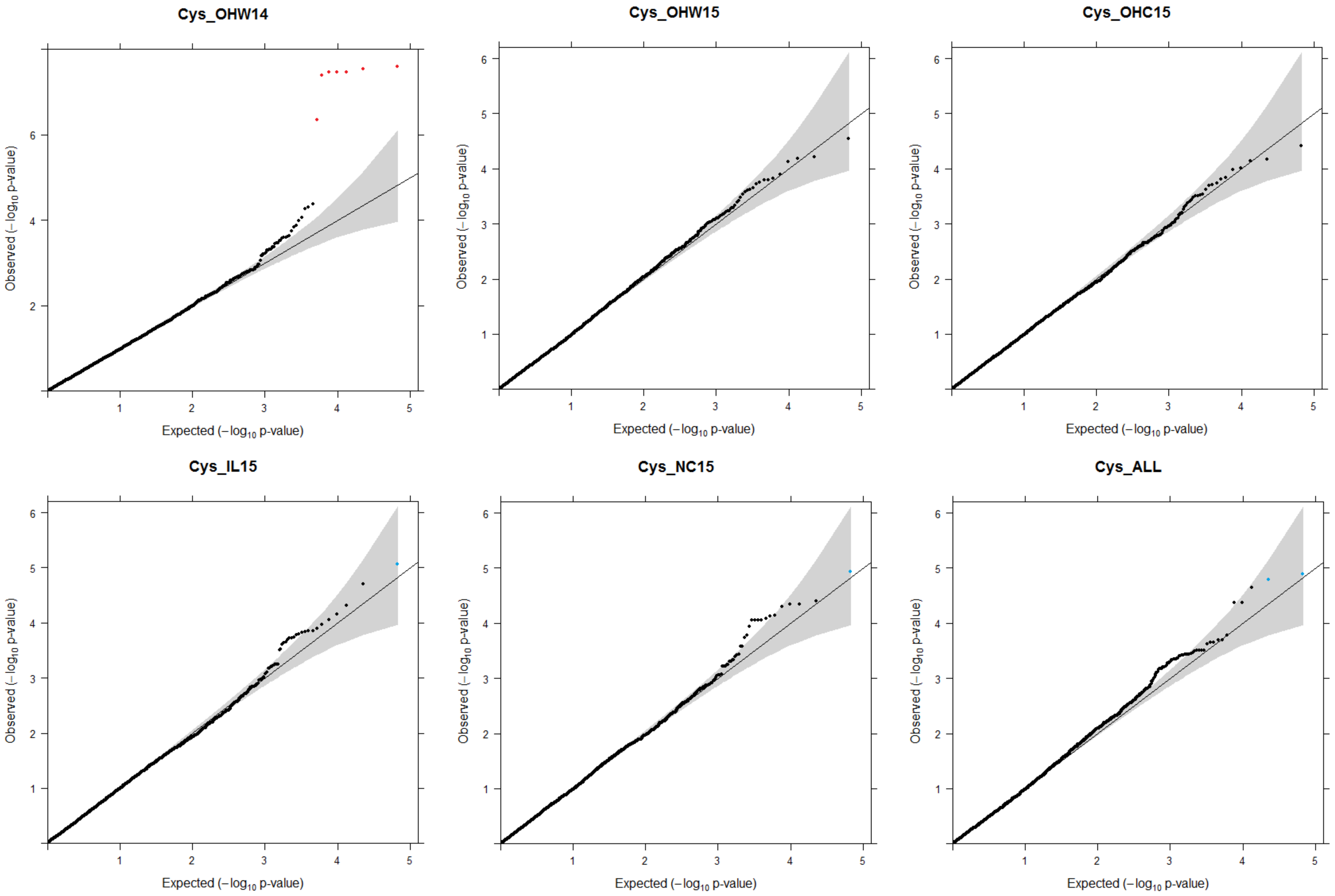

Fig. S15 QQ-plots for GWAS of 621 soybean accessions for Cys using CMLM. 34,014 SNPs were plotted by each environment and all environments combined. Red and blue dots represent the SNPs significantly associated at genome-wide significant threshold (5%) and suggestive significance thresholds (25%), respectively. Shaded region of the QQ-plots represent a 95% confidence interval.

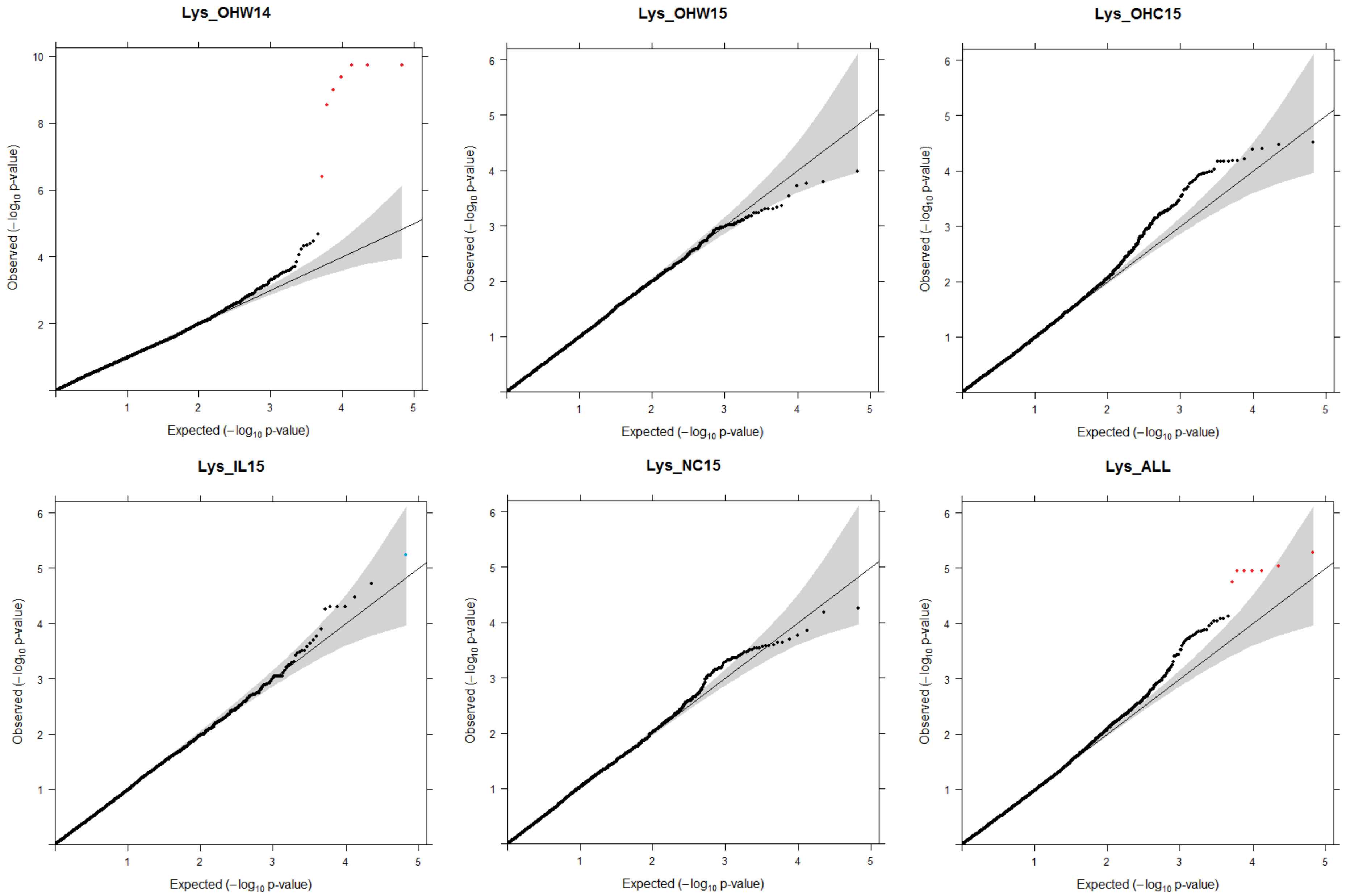

Fig. S16 QQ-plots for GWAS of 621 soybean accessions for Lys using CMLM. 34,014 SNPs were plotted by each environment and all environments combined. Red and blue dots represent the SNPs significantly associated at genome-wide significant threshold (5%) and suggestive significance thresholds (25%), respectively. Shaded region of the QQ-plots represent a 95% confidence interval.

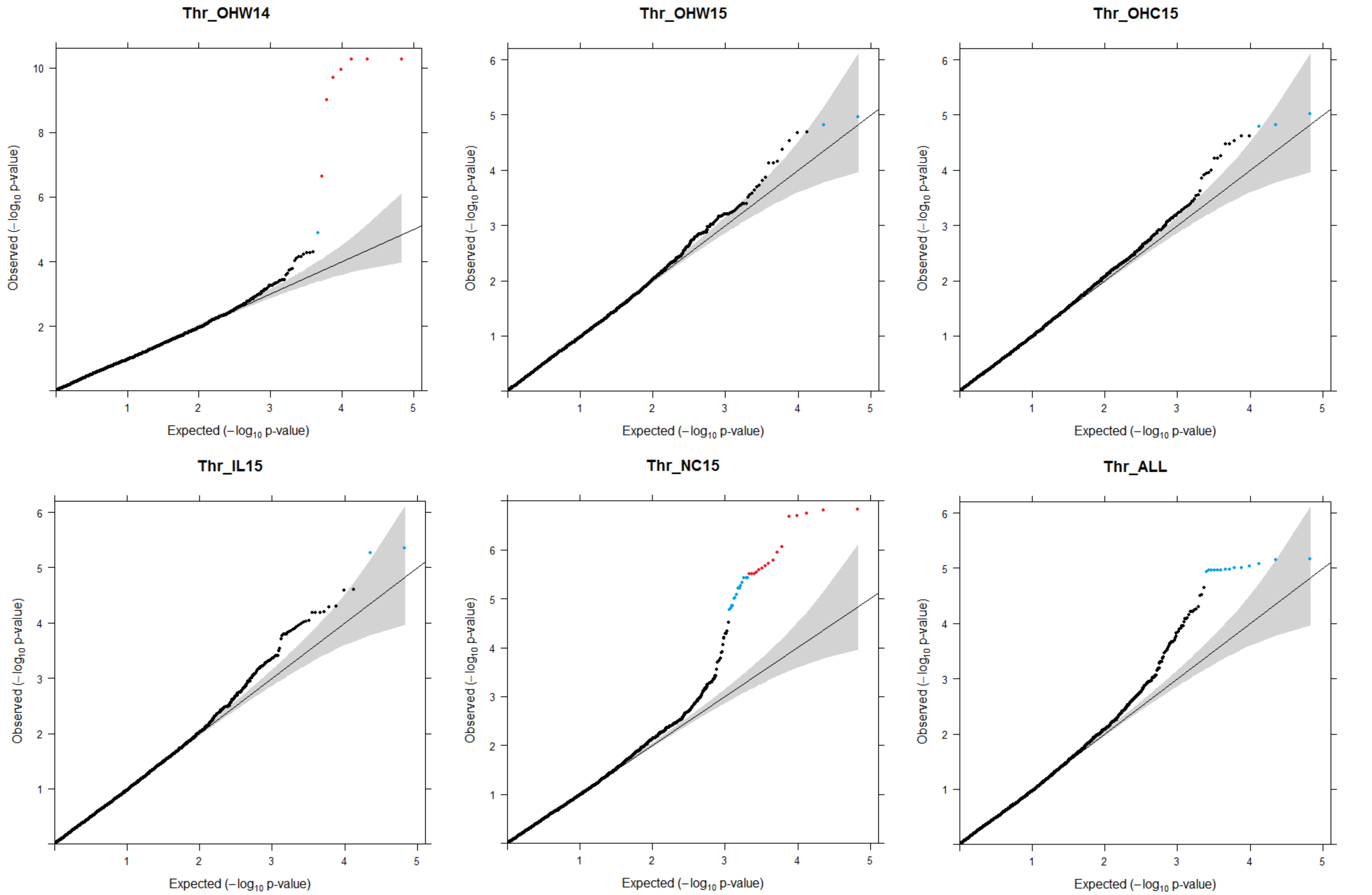

Fig. S17 QQ-plots for GWAS of 621 soybean accessions for Thr using CMLM. 34,014 SNPs were plotted by each environment and all environments combined. Red and blue dots represent the SNPs significantly associated at genome-wide significant threshold (5%) and suggestive significance thresholds (25%), respectively. Shaded region of the QQ-plots represent a 95% confidence interval.

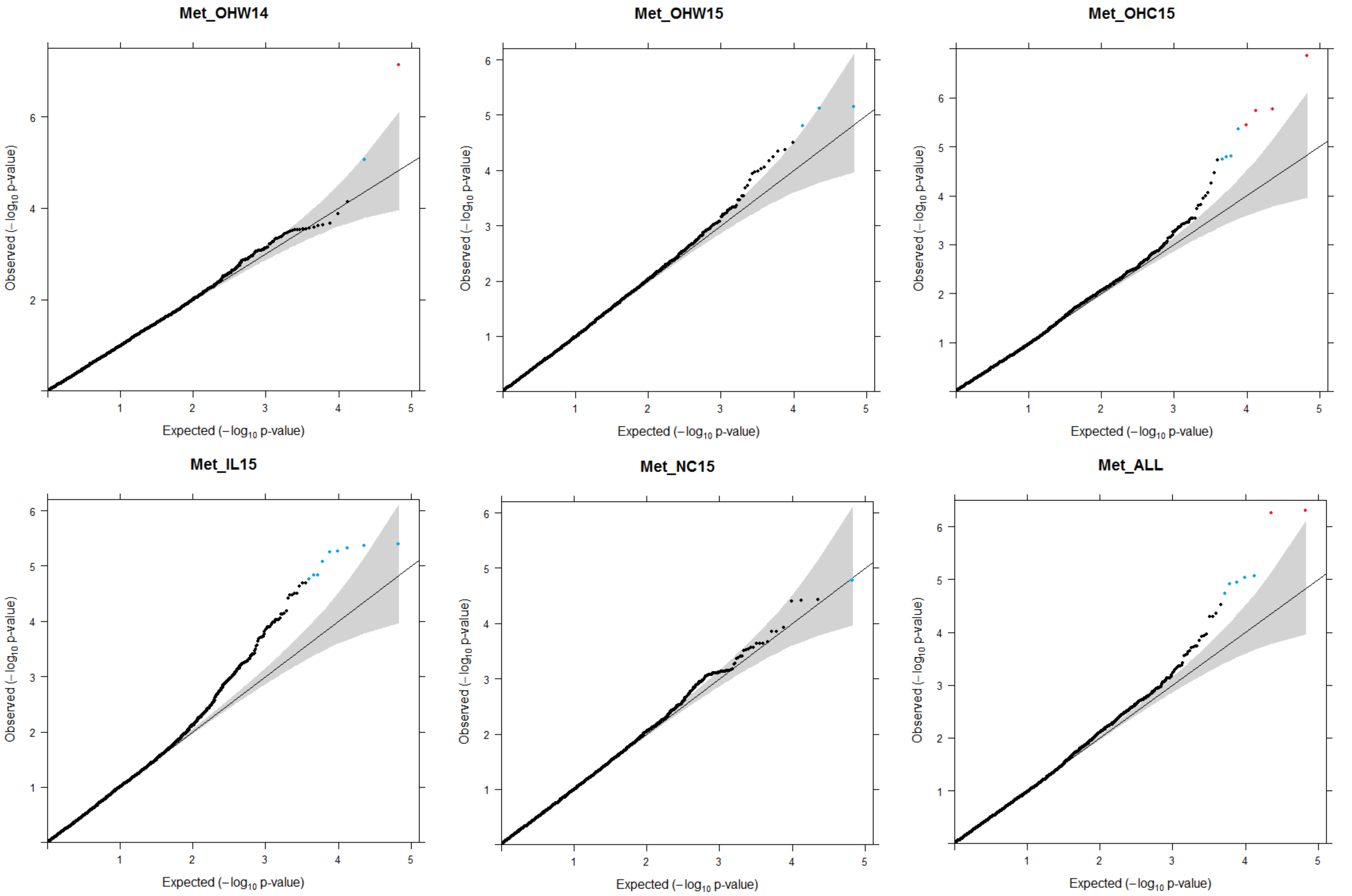

Fig. S18 QQ-plots for GWAS of 621 soybean accessions for Met using MLMM. 34,014 SNPs were plotted by each environment and all environments combined. Red and blue dots represent the SNPs significantly associated at genome-wide significant threshold (5%) and suggestive significance thresholds (25%), respectively. Shaded region of the QQ-plots represent a 95% confidence interval.

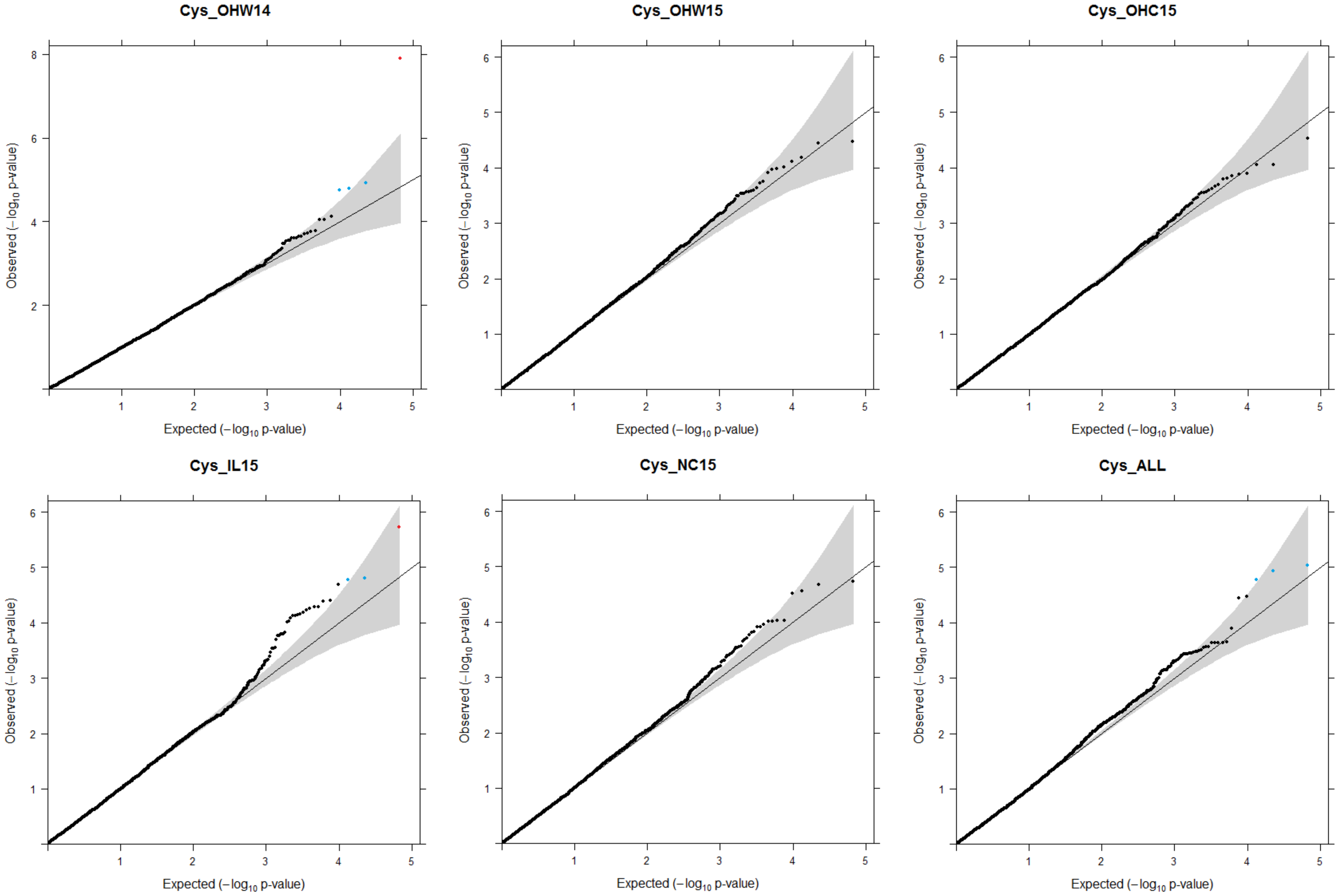

Fig. S19 QQ-plots for GWAS of 621 soybean accessions for Cys using MLMM. 34,014 SNPs were plotted by each environment and all environments combined. Red and blue dots represent the SNPs significantly associated at genome-wide significant threshold (5%) and suggestive significance thresholds (25%), respectively. Shaded region of the QQ-plots represent a 95% confidence interval.

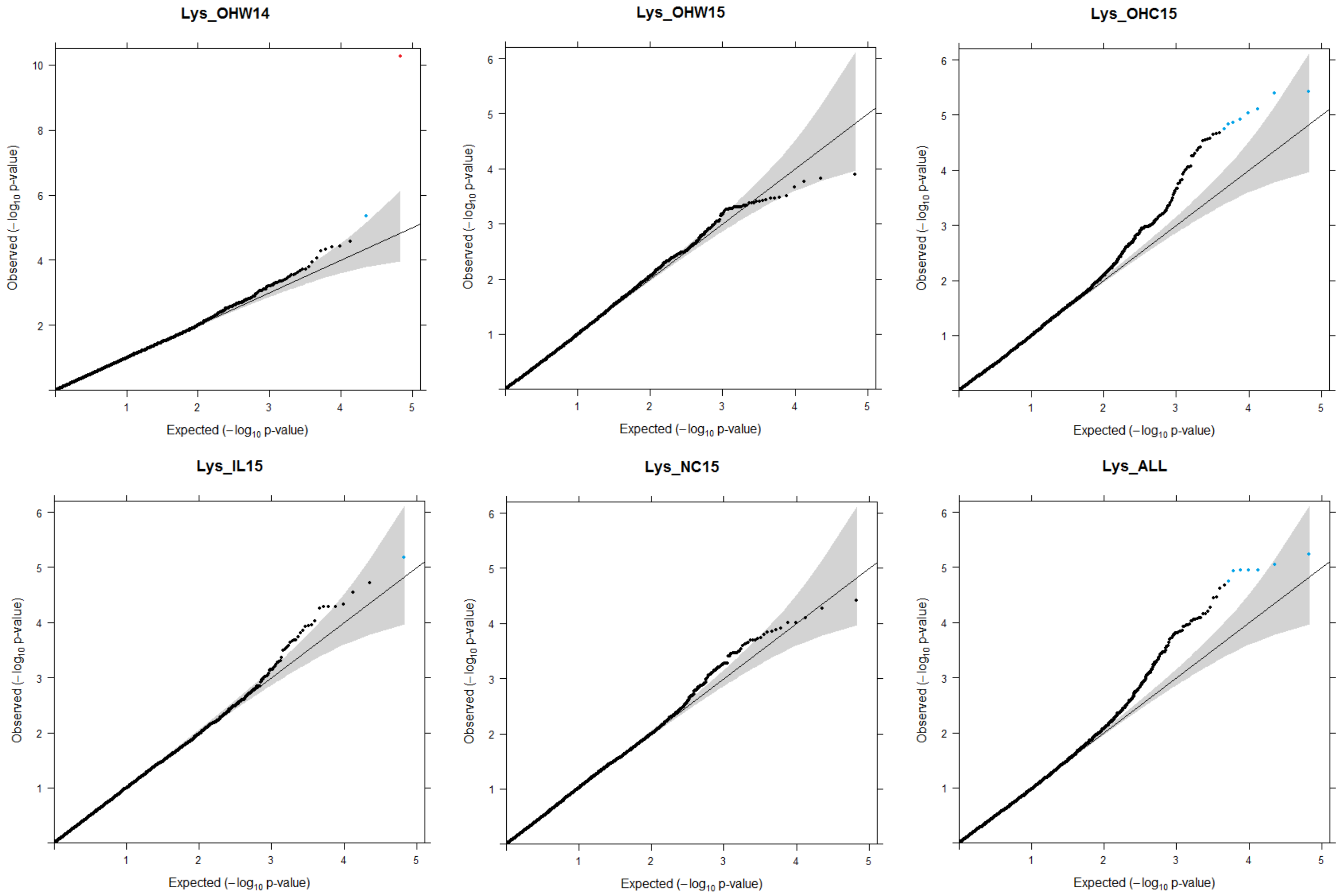

Fig. S20 QQ-plots for GWAS of 621 soybean accessions for Lys using MLMM. 34,014 SNPs were plotted by each environment and all environments combined. Red and blue dots represent the SNPs significantly associated at genome-wide significant threshold (5%) and suggestive significance thresholds (25%), respectively. Shaded region of the QQ-plots represent a 95% confidence interval.

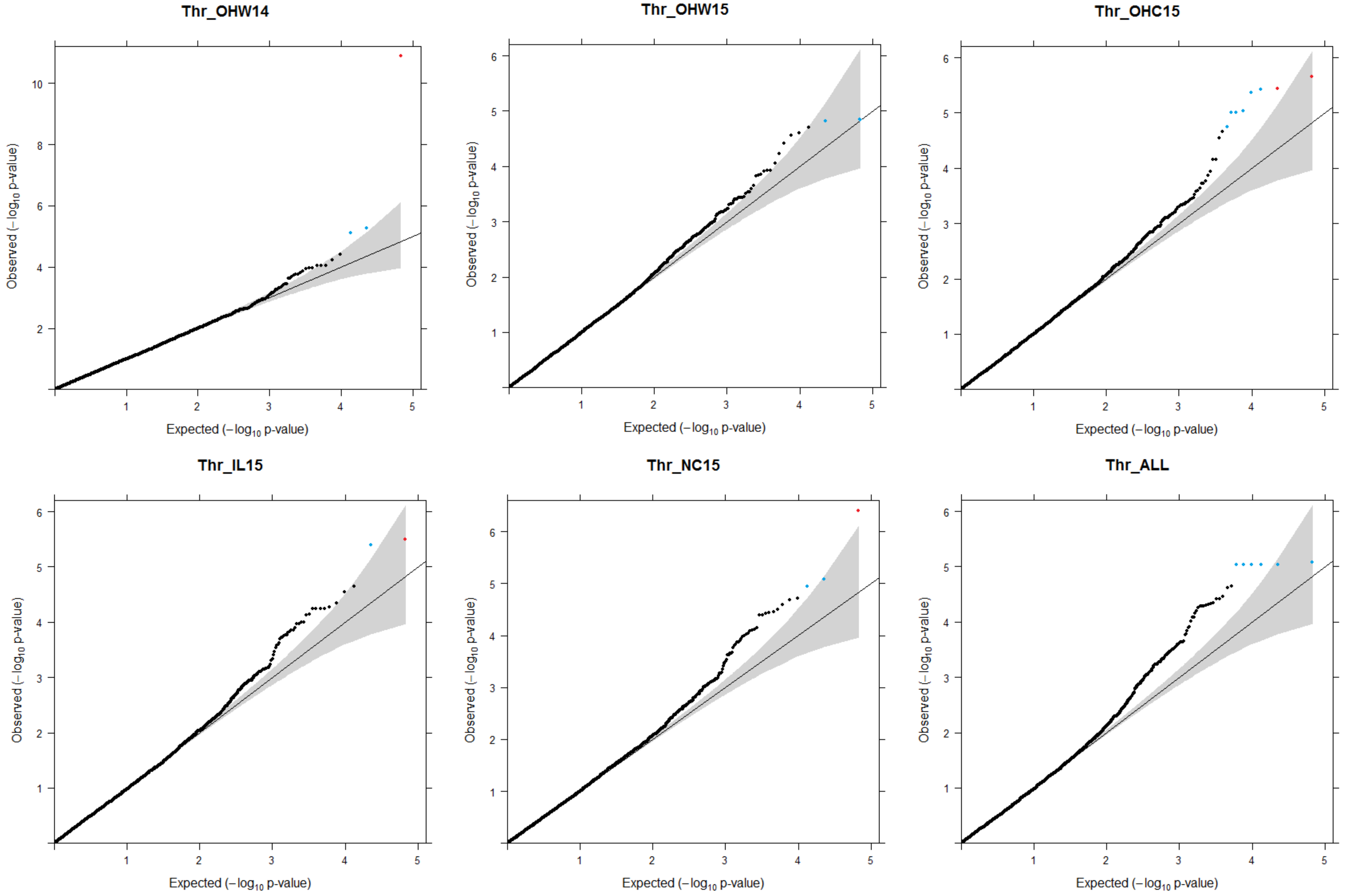

Fig. S21 QQ-plots for GWAS of 621 soybean accessions for Thr using MLMM. 34,014 SNPs were plotted by each environment and all environments combined. Red and blue dots represent the SNPs significantly associated at genome-wide significant threshold (5%) and suggestive significance thresholds (25%), respectively. Shaded region of the QQ-plots represent a 95% confidence interval.

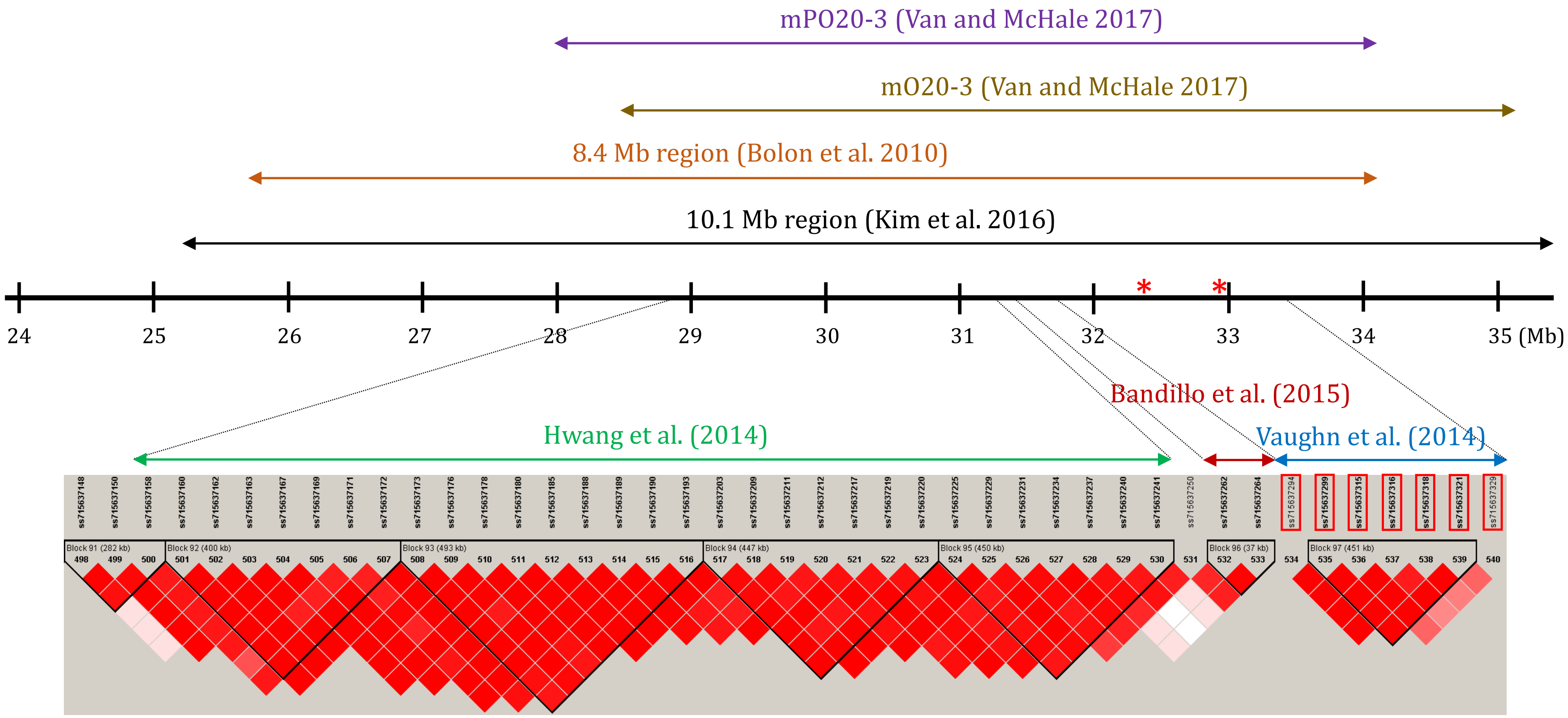

Fig. S22 The LD blocks from Chr 20 which were significantly associated with seed protein and oil contents (current study) compared with the genomic regions from six previous studies (Bandillo et al. 2015; Bolon et al. 2010; Hwang et al. 2014; Kim et al. 2016; Van and McHale 2017; Vaughn et al. 2014). LD blocks were defined by four-gamete method. Significant SNPs of the current study were indicated by red box. Asterisks represent the putative positional candidate genes mentioned in the main text.
